# Mitochondrial abnormalities as a target of intervention in acute myeloid leukemia

**DOI:** 10.3389/fonc.2024.1532857

**Published:** 2025-01-20

**Authors:** Elissa Tjahjono, Megan R. Daneman, Bernadetta Meika, Alexey V. Revtovich, Natalia V. Kirienko

**Affiliations:** Department of BioSciences, Rice University, Houston, TX, United States

**Keywords:** acute myeloid leukemia, leukemic stem cells, mitochondria, oxidative phosphorylation, mitophagy, glutaminolysis

## Abstract

Acute myeloid leukemia (AML) is an aggressive hematological malignancy; it is the most common acute leukemia in adults. AML prognosis is often poor, and relapse often occurs after initial remission. Recurrent genetic abnormalities underlying this disease and the presence of leukemic stem cells complicate disease treatment. However, the complex metabolic reprogramming that enables the unrestrained cell growth seen in these cells may also be their Achilles’ heel. In these cells, mitophagy operates as a double-edged sword. On one hand, it provides a source of building blocks for further cell division and serves as a method for removing damaged organelles, promoting cell survival. However, the profound metabolic changes to mitochondria also render these organelles more sensitive to damage and place them precariously close to excess mitophagic activation. This review discusses the dual role mitophagy plays in AML survival, the importance of targeting mitophagy to treat AML, and current progress in the area. The discovery and mechanism of action of multiple compounds that were used to inhibit or stimulate mitophagy and their effects on AML survival are also described. Further, we explore the combination strategy of mitophagy-targeting compounds with existing and/or novel chemotherapeutics to eradicate AML and discuss strategies to uncover new drug targets and novel mitochondria-targeting drugs.

## 1 Introduction

Leukemias are a group of hematological cancers characterized by the abnormal proliferation of partially-differentiated hematopoietic stem cells (HSCs) (1). Although there are multiple types of leukemia, the four most common are acute and chronic myeloid leukemias (AML and CML) and acute and chronic lymphocytic leukemias (ALL and CLL). Data from the National Cancer Institute (NCI) indicate that the five-year survival rate for patients is 70.6% for CML, 71.3% for ALL, and 88% for CLL. This contrasts with AML, where it is only 31.9%. AML survival negatively correlates with patient age and the presence of comorbidities; unsurprisingly, both conditions also reduce treatment options. AML is also characterized by particularly aggressive progression; if left untreated, patients can succumb to disease within weeks.

As can be inferred from their names, different blast cell populations are observed in different leukemias. AML shows excess myeloblast proliferation, which compromises normal signaling and differentiation, resulting in the accumulation of an immature and poorly functioning population of cells. This compromises normal hematopoiesis, leading to anemia and immune dysfunction. This is demonstrated by the susceptibility to opportunistic diseases (e.g., bacterial infections) in AML patients. Ultimately, AML patients succumb to some combination of leukostasis (a condition characterized by extremely elevated blast count and decreased oxygen flow to tissues, possibly due to blasts preventing erythrocyte flow), organ failure, and/or infection by common pathogens that are normally cleared by the immune system.

In most cases, AML blasts exhibit profound metabolic and mitochondrial changes that promote unrestrained cell division (discussed further in Section 2). The metabolic reprogramming and mitochondrial dysfunction seen in AML sensitize the cells to mitochondrial damage, which has recently sparked considerable interest in targeting mitochondria for therapeutic effect (2–5). For example, drugs that disrupt mitochondrial homeostasis by inducing or inhibiting mitophagy (autophagic degradation of mitochondria) are one area of particular interest (discussed further in Section 3) (6, 7).

The current standard first-line treatment regimen for AML is called induction and consolidation therapy. Its purpose is to reduce the number of leukemic blast cells in the body (de-bulk). During induction, patients are treated with a high dose of the cytosine nucleoside analog cytarabine (araC) in combination with an anthracycline (such as daunorubicin, doxorubicin, or idarubicin) that intercalates into DNA to disrupt topoisomerase II function. Together, these drugs induce profound cell death in proliferating myeloblasts in the bone marrow and blood (8). If induction is successful, patients are brought to a state called remission, clinically defined as less than 5% circulating leukemic blasts and recovery of normal hematopoiesis (8). De-bulking the leukemic blast cells often allows the body to temporarily regain hematopoietic homeostasis.

Once remission is achieved, induction is followed by consolidation. During this stage, patients are usually treated with a high dose of araC to prevent further myeloblast proliferation (or, preferably, to further reduce the number of leukemic cells). In some cases, consolidation is not done with araC but with other drugs selected to target specific AML mutations (1, 8, 9). Without consolidation, patients often experience rapid relapse because induction is ineffective against slowly cycling leukemic stem cells (LSCs), which co-exist in the bone marrow niche with their leukemic blast progeny and normal HSCs (10). Interestingly, available evidence indicates that LSCs are derived from partially differentiated HSCs that have aberrantly regained self-renewal capacity (10). LSCs that have not succumbed to treatment resume producing rapidly-cycling myeloblasts, promptly restoring the leukemic state.

Irradiation is another AML treatment, often used following induction and consolidation, to destroy bone marrow and nucleated blood cells, whether cancerous or not (11–13). Once these cell populations have been removed, an allogenic hematopoietic stem cell transplant (allo-HSCT) is performed to provide the patient with a functional population of bone marrow cells (8). Patients commonly receive maintenance chemotherapy after allo-HSCT, particularly those with a high risk of relapse. In some cases, this aggressive course of treatment is sufficient to abolish the leukemic cells and restore patients to long-term health. In many cases, however, this aggressive treatment leads to leukemia cells that are resistant to the treatments being used (11).

Relapsed and refractory AML, which are indicative of treatment failure, are primarily caused by drug resistance. The emergence of treatment-resistant clonal lines in patients is another cause for low overall AML patient survival. There are multiple mechanisms of cancer drug resistance, including aberrant expression of drug-resistance proteins and microRNAs, dysregulation of signaling pathways, and often the same genetic alterations that caused the illness in the first place (14–17). Many of these dysregulations implicate mitochondrial signaling and metabolic pathways. Other resistance mechanisms include modifying or preventing incorporation of the drug. For example, mutations in deoxycytidine kinase (dCk) activity reduce conversion of araC to a dideoxy form that can be incorporated into replicating DNA. AraC can also be converted into an alternate, unusable metabolite to prevent incorporation (18–20).

Resistance in AML is often driven by decreased import and increased efflux (21). Alterations to import and efflux are common mechanisms of resistance observed for almost all FDA-approved treatments, including anthracyclines, mitoxantrone, etoposide, and methotrexate (22–26). For example, increased expression of the drug efflux pump, P-glycoprotein (Pgp/ABCB1), is associated with poorer prognosis (27).

Mutations in surface antigens can also confer resistance. For example, CD33 is a myeloid marker expressed in about 90% of AML patient cells, but rarely expressed on normal HSCs (28). The antibody-drug conjugate gemtuzumab ozogamicin (GO), which is comprised of an anti-CD33 monoclonal antibody fused to the antitumor antibiotic calicheamicin, shows resistance linked to the activity of glycogen synthase kinase-3 (GSK3). The effectiveness of GO decreases with increased activity of GSK3. Such resistance has been overcome with parallel use of GSK3 inhibitors in cell lines (29). Although GSK3 is tightly linked to a wide variety of mitochondrial functions, even in cancer cells, GSK3-mediated resistance to GO was attributed to reduced CD33 expression (reducing the efficacy of antibody targeting), increased lysosomal drug degradation, increased export, and increased expression of the anti-apoptotic protein BCL-2 (29, 30).

In still other cases, resistance to therapies is caused by further changes to mitochondrial metabolism that reduce treatment effectiveness. Examples of this include upregulating the anti-apoptotic protein BCL-2, upregulating amino acid catabolism, and utilizing alternative energy pathways such as lipid oxidation (31–33). This review will discuss recent findings and progress in leukemia treatment, particularly focusing on the importance of targeting the mitochondria using changes in mitophagy to bolster AML treatment.

## 2 Metabolic reprogramming in AML

### 2.1 Changes in oxidative phosphorylation

Most AML cells have mutations in one or more genes that affect cell growth or metabolic function and anomalies in mitochondrial metabolism are frequently observed (3, 4, 34–37). Like all cancer cells, leukemic cells need to increase their energy production to support their increased cell division (5, 38). For many cancer types, this drive for increased energy induces so-called Warburg metabolism, which involves upregulation of glycolysis and reduced OXPHOS (39). Contrastingly, AML blasts instead appear to show an increase in dependency on OXPHOS compared to most other cancer types (2).

One common cause of the increased OXPHOS in AML is mutation of the membrane-bound receptor Fms-like tyrosine kinase 3 (FLT3). Normal FLT3 activity is associated with regular hematological cell growth and development (40). But internal tandem duplications of the kinase domain (known as FLT3-ITD, found in about 20% of AML cases) and point mutations or deletions in the kinase domain (FLT3-TKD, present in about 10% of AML cases) have been shown to disrupt Ras signaling and upregulate expression of PDP1, a key regulator of the pyruvate dehydrogenase complex (41, 42). This increases pyruvate flux through the citrate cycle, increasing OXPHOS activity, energy production, and ROS generation.

Several inhibitors of FLT3, such as midostaurin, gilteritinib, quizartinib, or sorafenib, have been identified and tested for use in AML patients with FLT3 mutations (43). Unfortunately, FLT3 inhibitor monotherapy is frequently unsuccessful and often results in relapse and resistance (44). Combining various FLT3 inhibitors with commercial chemotherapeutics has been attempted in clinical trials, with mixed results (45). Notably, the addition of midostaurin to induction and consolidation treatment demonstrated an overall survival benefit in the clinical trial, RATIFY, and was approved by the FDA (9, 46–48). FLT3 inhibitors have had heterogenous success as maintenance therapy after allo-HSCT, thus further investigation into relapse-free cases is warranted (9, 47, 49–51).

Mutations in isocitrate dehydrogenase (IDH, which are found in about 20% of AML patients) are associated with increased mitochondrial oxidative metabolism, including Complex I activity, respiration, and fatty acid oxidation. IDH inhibitors, such as ivosidenib or enasidenib, were evaluated and showed patterns similar to FLT3 inhibitors: monotherapy often showed temporary clinical utility, but was followed by the rapid rise of resistance (52–54). Combination with other therapies was roughly twice as effective, but relapse and resistance were still common, making patients with mutations in IDH a key population that urgently needs useful therapies. Disruptions of IDH and FLT3 not only support AML cell survival, they can also promote resistance to chemotherapy (55–57).

Although AML cells consume more oxygen at the basal metabolic level, the efficiency of ATP generation does not improve because the cells exhibit significant proton leak and poor coupling efficiency (ratio of oxygen consumed for ATP production to oxygen lost in proton leak) (36). OXPHOS is driven by the proton gradient generated by the electron transport chain (ETC) pumping protons from the mitochondrial matrix to the intermembrane space. Some protons pass back across the mitochondrial membrane without contributing towards ATP production (i.e., leak) (58–61). OXPHOS dependency increases the level of reactive oxygen species (ROS) making AML cells more sensitive to ROS levels (61). AML blasts also have reduced spare reserve capacity, which is the difference between their basal metabolic load and their maximum metabolic rate. This increase in OXPHOS drives parallel increases in ROS production and accumulation of mitochondrial damage, which is likely a cause for the observations that AML blasts have a reduced capacity to tolerate exogenous oxidative stress (34, 35). For example, while studying responses of the NCI-60 cell panel to a variety of drugs, we observed that leukemic cells show particular sensitivity to mitochondrial damage (3). Further analysis demonstrated that, although they have nearly twice as many mitochondria as healthy hematopoietic cells, AML cells have poor respiration capacity and coupling efficiency. This also led us to believe that mitochondrial damage may be a viable adjuvant to other chemotherapies.

These mitochondrial changes are likely one reason that AML blasts show increased mitochondrial mass and increased rates of mitochondrial biogenesis (34). Other reports have shown that blasts also exhibit a greater dependence upon fatty acid oxidation (37), particularly very long-chain fatty acid oxidation (62). Alteration of sphingolipid composition, specifically reduction of ceramide, also leads to increased mitochondrial biogenesis and OXPHOS (63). This mitochondrial remodeling was found to contribute to AML cells’ resistance to the combination of daunorubicin and araC, and standalone doxorubicin treatment (63, 64). Similarly, these cells are sensitive to treatment with ceramides, particularly when combined with an OXPHOS inhibitor (35). Upon treatment with the combination of C_6_-ceramide and tamoxifen, mitochondria were seen to colocalize with autophagosomes, indicating activation of autophagy and mitophagy (65). Remarkably, exposure to either drug alone had no apparent effect on AML cell viability, but the combination resulted in a strong additive effect. One possible explanation for this outcome is that the mitochondrial damage that accumulates during AML overtaxes mitochondrial recycling pathways, and further stimulation tips the cell into activation of programmed cell death pathways. This supports the idea that mitophagy may be used to eliminate treatment-resistant AML cells (see Section 3).

Although it is well-recognized that OXPHOS activity increases ROS production, surprisingly, inhibiting OXPHOS also induces the production of ROS in AML cells (66, 67). For example, treatment of OCI-AML3 cells with the OXPHOS inhibitor IACS-010759 alone, or in combination with low-dose araC, significantly increased ROS production (67). Increased ROS levels after treatment with IACS-010759 and araC also trigger mitochondrial fission that leads to mitophagy. This combination of mitochondrial activities induces the resistance of AML to OXPHOS inhibition by promoting mitochondrial transfer from mesenchymal cells and, unfortunately, renders this combination therapy relatively ineffective (67). Nevertheless, OXPHOS inhibitors and other mitochondria-targeting chemotherapeutics are widely used to improve treatment response (68).

### 2.2 Increased glutamine utilization in AML

Another significant example of metabolic reprogramming in AML cells is the highly increased use of glutaminolysis by blasts and LSCs, so that they are occasionally referred to as glutamine-addicted leukemia (69–72). AML cells rely on glutamine as a carbon source for energy production and its derivative, glutathione, for controlling ROS. Glutamine is transported into the cell, broken down into glutamate, and oxidized into α-ketoglutarate before entering the citric acid cycle (CAC) to produce energy and bypass the need for glucose in energy production (73). Additionally, the direct utilization of α-ketoglutarate bypasses the rate-limiting step of CAC: converting isocitrate into α-ketoglutarate (74). The products of the CAC are used to drive OXPHOS by activating the complexes of the ETC to create the proton gradient for ATP synthesis. The utilization of glutamine over glucose increases energy production via OXPHOS, making glutaminolysis a promising therapeutic target.

Drugs targeting glutamine uptake, glutamine antagonists, glutaminase inhibitors, and asparaginases are all under investigation (6). Targeting glutaminolysis decreases OXPHOS capacity and inhibits the CAC cycle. However, these compounds are also subject to resistance. Leukemic cells can be rescued by excess CAC intermediates derived from glutaminolysis, and acquire chemoresistance through metabolic shifts to favor other carbon sources such as glucose (75, 76). Additionally, immune cells rely on glutaminolysis to generate excess energy, resulting in toxic side effects in some cases (77). Therefore, additional characterization of glutaminolysis is needed to generate more specific and less toxic metabolism-targeting compounds.

Glutamine metabolism also provides building blocks for glutathione, which reduces cellular ROS (78). Inhibiting the enzymes responsible for converting oxidized glutathione (GSSG) to its reduced form (GSH) increased ROS levels and killed cells (79, 80). GSH is therefore also a potential therapeutic target (81).

### 2.3 Additional metabolic reprogramming in LSCs and the bone marrow microenvironment

LSCs also exhibit considerable metabolic reprogramming. These cells co-exist in the bone marrow microenvironment with blast cells and more typical HSCs, but LSCs are thought to be differentiated from normal HSCs by the heterogenous expression of a variety of surface markers, including CD32, CD44, CD45RA, CD47, CD96, CD123, and TIM3 (10, 82). Unlike HSCs, which divide and differentiate at a much slower rate and can rely on glycolysis and the limited OXPHOS possible in the bone marrow niche for their energy needs (10), LSCs divide more rapidly, generating a higher energetic demand compared to HSCs. LSCs fulfill this need for energy by increasing OXPHOS and mitochondrial mass, like AML and CML blasts (10, 82, 83). As the bone marrow microenvironment is comparatively hypoxic, LSCs compensate this condition by increasing lipid oxidation and glutaminolysis to support this requirement (10, 32, 82). AML blast cells and LSCs cultured under hypoxic conditions have been shown to upregulate both general macroautophagy and mitophagy to support development and mitigate cellular damage (84, 85). Activation of these pathways helps to prevent build-up of ROS that may induce apoptosis and is likely to be a key factor in the low levels of ROS observed in LSCs (86).

The bone marrow microenvironment has also been shown to protect LSCs and leukemic blast cells from chemotherapeutics. Bone marrow stromal cells transfer mitochondria to AML blasts via leukemia-derived tunneling nanotubes, promoting chemoresistance (66, 67). A study involving a niche-like coculture system showed that up to 14% of the mitochondria in stromal cells are transferred to AML cells with which they are in physical contact, leading to a 50% increase in mitochondrial ATP production (87). Organelle transfer is also stimulated by superoxide production by AML-derived NADPH oxidases (NOX2), which increase ROS in the bone marrow stromal cells (66, 67). Transferred mitochondria from bone marrow stromal cells not only contributes to ATP generation via the CAC and OXPHOS, but also to production of GSH, which is likely to ameliorate the ROS and help limit oxidative crisis (88).

LSCs also exhibit an increase in mitochondrial number but a reduction in size due to increased rates of mitochondrial fission (86, 89). The AMPK/FIS1 signaling pathway is constitutively expressed in AML LSCs, promoting fission, activating mitophagy, and sustaining proliferation. This activity has also been theorized to assist in promoting stemness in the LSCs (86, 89). Consistent with this, loss of FIS1 expression leads to upregulation of hematopoietic and myeloid differentiation markers (86). A similar phenotype is also observed after loss of mitophagic regulators (PINK1, TBC1D15, etc.), indicating that mitochondrial fission and mitophagy are critical for the maintenance of LSC viability (86).

## 3 Mitophagy in AML

### 3.1 Mechanisms of mitochondrial autophagic degradation

Autophagy, or self-eating, is a pro-survival mechanism that allows cells to recycle large portions of their machinery, ranging from portions of the cytoplasm and its protein components to whole organelles. Cellular material is engulfed into a membrane-bound compartment called an autophagosome that then fuses with lysosomes to enable the degradation of the cellular materials. Recycling of organelles and other cellular structures decomposes them into their constituents, which can then be used to replenish necessary metabolites or to generate new organelles (86). Autophagy is often triggered by changes in cellular environment, particularly nutrient deprivation, oxidative stress, or other stress signals. Autophagy is also frequently upregulated in cancer cells, including AML, where it is thought to provide raw materials (e.g., lipids and amino acids) to support cell division (90–93).

Autophagic degradation is also used as a selective mechanism for recycling damaged or dysfunctional mitochondria (known as mitophagy). Mitochondria absorb damage from a variety of causes, including external, stress-inducing environmental stimuli (e.g., carcinogens, pesticides, and heavy metals) and the internal metabolic processes that produce ATP (94–97). These factors damage the proteins, lipids, and DNA of the organelle, reducing functionality. Worse, this damage often decreases OXPHOS efficiency, further increasing ROS levels in a vicious, downward cycle. Mitochondria damaged in this way are tagged by pro-mitophagic proteins that facilitate autophagosome recruitment and mitochondrial recycling (see below). Cells have relatively few mitochondrial repair pathways; therefore, they heavily rely on mitophagic turnover for resolving damage (98, 99).

**Figure 1:**
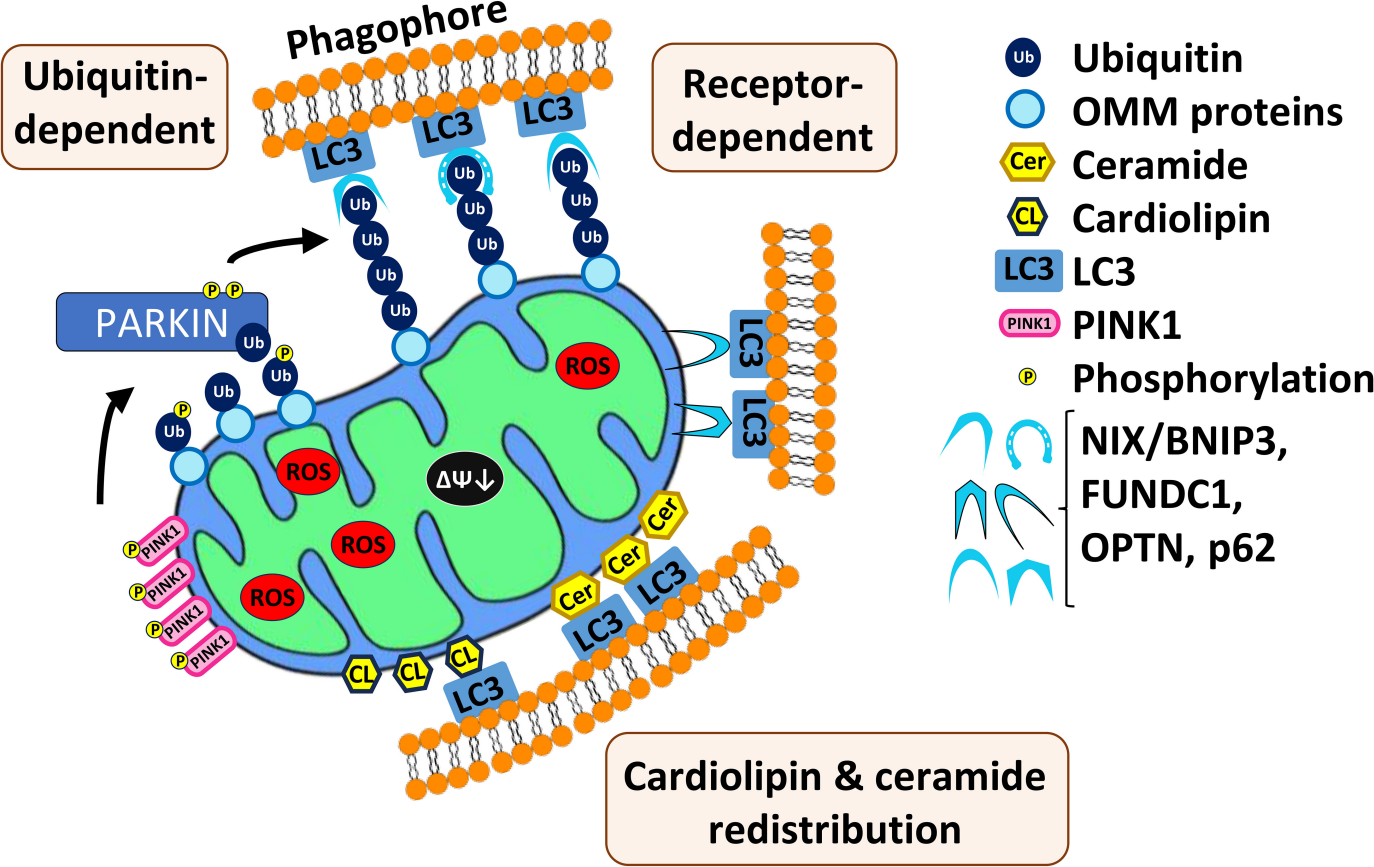
Three main mechanisms of mitophagy in mammals. First, mitochondrial depolarization and impair of protein import cause accumulation of PINK1 on the outer membrane of mitochondria (OMM). PINK1 auto- and transphosphorylates and other targets such as Parkin, which then catalyzes transfer of ubiquitin to various mitochondrial-resident transmembrane proteins. This allows recognition by the autophagosomal sequestration machinery. Second, multiple mitophagic receptors (NIX/BNIPS, FUNDC1, OPTN, p626) are also able to recognize the polyubiquitinated substrates and recruit autophagic machinery. Third, relocalization of cardiolipin and ceramide to the outer membrane of mitochondria where they interact with autophagic machinery.

Two main mechanisms of mitophagy have been identified in mammals (**Figure 1**), and both have been linked to carcinogenesis and have been implicated in the progression and poor prognosis of human AML (100, 101). The first mechanism is regulated by the well-known PTEN1-induced kinase (PINK1)-Parkin pathway, which has been subjected to more study than the other known pathways. Under normal circumstances, PINK1 is localized to the outer membrane of mitochondria. Normal mitochondrial import processes bring the protein into the organelle, where it is cleaved by PARL, a protease resident in the mitochondrial intermembrane space. The remaining portion of PINK1 returns to the cytosol where it is degraded by a proteasome (102). If mitochondrial membrane potential is lost (i.e., the mitochondrial membrane is depolarized) or protein import is impaired for some other reason, PINK1 accumulates on the outer mitochondrial membrane. Under these conditions, PINK1 is auto- and transphosphorylated, licensing the phosphorylation of additional targets, such as the ubiquitin ligase Parkin (**Figure 1**). Once activated, Parkin catalyzes the transfer of ubiquitin to various mitochondria-resident transmembrane proteins, allowing them to be recognized by autophagosomal sequestration machinery, such as the protein MAP1-LC3 (103, 104). Ubiquitination of outer mitochondrial membrane proteins facilitates their recognition by additional receptors, such as NIX (NIP3-like protein X), BNIP-3 (BCL-2/adenovirus E1B 19 kDa interacting protein 3), SQSTM1 (sequestosome 1)/p62 (105), FUNDC1, or OPTN (optineurin).

Mitophagy can also be induced by redistribution of lipid species (**Figure 1**). For example, cardiolipin is a phospholipid found in the inner mitochondrial membrane (106). During mitophagy, cardiolipin is resorted to the outer mitochondrial membrane and may play a role in recognition of damaged mitochondria and recruitment of the autophagosome (107). Additionally, C_18_-ceramide, a key component of sphingomyelin, which is a major plasma membrane lipid, has been associated with mitophagy (65). Depletion of C_18_-ceramide by genetic disruption of the enzyme responsible for its biosynthesis, CerS1, compromised sodium selenite-mediated mitophagy. Additionally, C_18_-ceramide was shown to relocalize from the endoplasmic reticulum to the outer membrane of mitochondria where, like cardiolipin, it interacts with MAP1-LC3 (65). Cardiolipin and CerS1/C_18_-ceramide synthesis are associated with tumor suppression, therefore their lack of expression facilitates AML tumorigenesis (65, 108, 109).

Mitophagy can serve as a pro-survival pathway for AML cells by eliminating damaged mitochondria, reducing signaling that may trigger programmed cell death pathways (110, 111). Consistent with this, high expression of mitophagic receptors, including *SQSTM1*, is associated with increased AML cell survival, contributing to poorer patient outcomes (100). This led to interest in mitophagic receptors as a target for clinical development. *SQSTM1* knockout decreased AML proliferation in multiple human and murine AML cell lines. Deletion of this gene also impairs myeloid leukemia progression and prolongs survival in mice (100). Another study similarly found that the downstream SQSTM1 protein (also called p62) and another mitophagic receptor, BNIP3L, increase leukemic cell survival in human AML (112). When bound to the outer membrane of damaged mitochondria, p62 and BNIP3L promote autophagy by recruiting autophagosomes (113). Knockdown of either protein compromised mitochondrial quality and sensitized AML cells to mitochondria-targeting therapeutics. When ROS levels become too high, LSCs induce mitophagy to limit ROS production, or else they will be pushed out of quiescence and cell death pathways will be activated (61, 86). However, high levels of mitophagy in LSCs and bulk AML cells induce multiple cell death pathways (see section 3.4) (65, 114). Therefore, mitophagy plays a dual role in therapeutic approaches: as a resistance mechanism and potential therapeutic target, requiring precise regulation and balance of mitophagy. For example, the pro-autophagy protein, p62, is also a downstream target the of survival and inflammatory pathway, NF-kB (115, 116). Activated p62 upregulates the anti-autophagy and pro-autophagy regulators, mTORC1 and NRF2, respectively. mTORC1 promotes cell growth, inhibits autophagy, and increases metabolism, including glycolysis and lipogenesis, whose upregulation has been implicated in driving carcinogenesis (117, 118). However, activating the antioxidant regulator, NRF2, induces mitophagy (119). This phenomenon highlights the tight regulation of mitophagy that leukemic cells undergo to survive.

In addition to metabolic dysfunction, mitochondria in AML cells also show abnormalities in their shape and structure. Depending on the genetic mutation in the AML cells, many ultrastructural parameters, including mitochondrial volume, the number and diameter of cristae, and the number of matrix granules vary (120). Unsurprisingly, these changes affect the cells’ respiratory profiles including ROS production, basal respiration, proton leak, ATP production, and spare respiratory capacity. Compared to other leukemic cell lines, OCI-AML3 (a representative AML cell line), had more mitochondria and larger cristae. Despite this, OCI-AML3 displayed comparatively lower oxygen consumption rate, indicating inefficient OXPHOS (120). Some studies have argued that the increased mitochondrial mass and number, along with low reserve capacity, a partially depolarized membrane, and the other problems observed, indicate that AML cells’ mitochondria are generally dysfunctional (4, 114). It is also speculated that the larger mitochondrial network observed may be required for other anabolic growth processes (4). Exploiting AML’s dysfunctional mitochondrial characteristics and the tight regulation of mitophagy is explored as a promising avenue for leukemia treatment.

These changes in mitochondrial structure also limit mitophagy, as mitochondria are generally too large to be effectively engulfed for autophagic degradation, so they must be reduced to smaller, more digestible pieces. This is one reason that effective mitophagy requires mitochondrial fission, a process in which proteins in the inner mitochondrial membrane disperse the organelle into smaller pieces. Mitochondrial fission has a counterpart, fusion, wherein smaller mitochondria merge. Fission and fusion are key to the maintenance of mitochondrial quality, as they allow damaged mitochondria to mix their contents to dilute the damage or, by mechanisms that remain unclear, segregate the damaged material into heavily damaged organelles for turnover. The most important proteins for mitochondrial fusion are MFN1 (Mitofusin 1), MFN2 (Mitofusin 2), and OPA1 (optic atrophy 1), while mitochondrial fission is mediated by the master regulator DRP1 (dynamin-related protein 1) and the DRP1-recruiting protein, FIS1 (118). Both DRP1 and FIS1 have been shown to regulate mitophagy as well (86, 121, 122).

### 3.2 Mitophagic inhibitors as treatment for AML

As noted above, mitophagy can remove damaged mitochondria, reduce ROS production, improve mitochondrial function, and provide building blocks for continued cell division. As such, it is a natural target for intervention in AML, and several studies have reported positive effects from preventing mitophagy.

The most notable of these involved XRK3F2, an anti-leukemic compound that exerts its effect by impairing the activity of SQSTM1/p62 (123). XRK3F2 treatment causes accumulation of p62 on the mitochondria but reduced colocalization of mitochondria with MAP1-LC3, suggesting that autophagosome recruitment had failed. Consequently, ROS was upregulated, and cell death was triggered. Most importantly, this cytotoxic effect is selective to LSC as neither murine nor human HSCs exhibited significant death after treatment with XRK3F2 (123).

**Figure 2:**
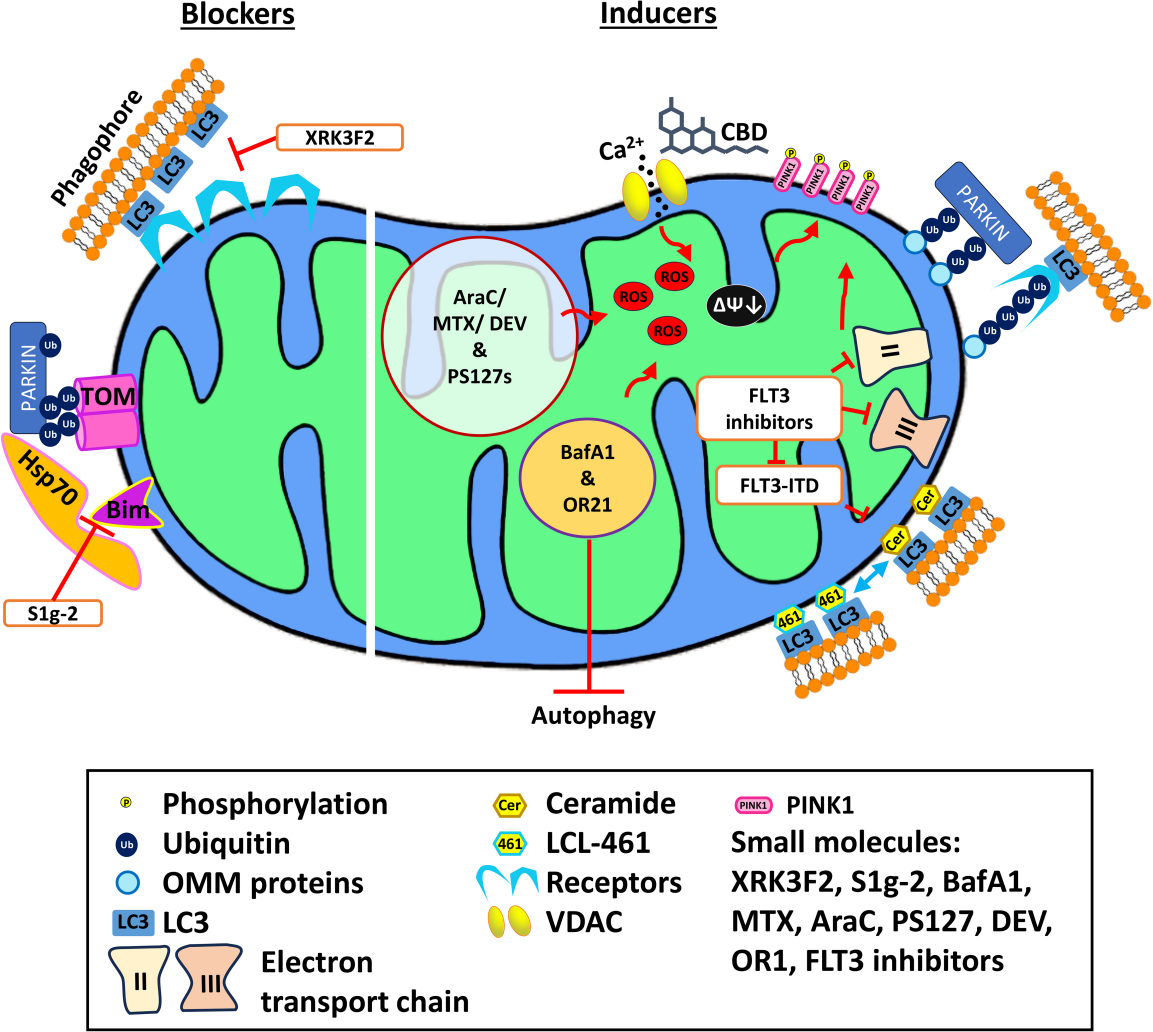
Mechanism of action of various mitophagy blockers and inducers. Mitophagy blockers include XRK3F2 and S1g-2, which binds to the ZZ domain of p62 and Hsp70, respectively, preventing mitophagy. Mitophagy inducers including cannabidiol, PS127 family compounds, BafA1, and OR21 exert their effect by inducing ROS production, thus activating mitophagy. Meanwhile, LCL-461 is a mitochondria-targeted ceramide analog that binds to LC3B and recruits autophagosomes. AraC, cytarabine; BafA1, Bafilomycin A1; DEV, devimistat; MTX, methotrexate; VDAC, voltage-dependent anion channel.

Another promising candidate is the small molecule, S1g-2 (124). This compound was originally discovered in a screen for novel inhibitors of Hsp70, a chaperone that is often overexpressed in drug-resistant CML cell lines (125). S1g-2’s mechanism of action involves binding to Hsp70 (**Figure 2**), disrupting its interaction with the Bcl-2-like protein Bim, and blocking the oncogenic activation of several proteins, including AKT, Raf-1, and eIF4e. Treatment with S1g-2 induced apoptosis and reduced *in vivo* burden of CML cells (125). Recently, it has been shown that under stress, the Hsp70-Bim protein complex translocates Parkin and TOMM20 together, leading to TOMM20 ubiquitination and subsequent mitophagic activation (124). Disrupting the interaction between Hsp70 and Bim with S1g-2 blocks mitophagy and indirectly activates apoptosis. As mitophagy is upregulated in both myeloid leukemias, the novel role of Hsp70-Bim in mediating mitophagy may extend S1g-2’s potential to use in AML as well.

### 3.3 Mitophagic activators as treatment for AML

Work in our lab to identify compounds that may stimulate mitophagy and improve chemotherapeutic treatment has focused on the PINK1-Parkin axis. We used a high-throughput, phenotypic screening in *Caenorhabditis elegans* to search for compounds that increased accumulation of a GFP-tagged *C. elegans* ortholog of PINK1 on mitochondria (126). One advantage of using this system is that it allows rapid removal of compounds that are biochemically effective but have substantial toxicity; dead worms generally do not produce GFP-labeled mitochondria. Using this approach, we identified several compounds that promote PINK-1 accumulation and stimulate mitophagy. The most effective compound and its analogs, called the PS127 family, induced ROS production and impaired mitochondrial respiration only in leukemic cells, and caused accumulation of mitophagic and necroptotic markers. They also activated apoptosis and ferroptosis. Finally, treatment increased survival of mice engrafted with human AML cells (114). The addition of the ROS scavenger, N-acetyl cysteine, significantly rescued PS127 family-induced cytotoxicity, indicating that AML cells are sensitive to increases in ROS. Other labs have also successfully explored whether increasing ROS could be an effective means for inducing leukemic cell death (2, 67, 127).

Increases in ROS can also result from the inhibition of ETC complexes, and by extension, OXPHOS. The ETC Complex I inhibitor, IACS-010759, was shown to increase mitochondrial fission and mitophagy upon treatment in AML cell lines (67). Interestingly, OXPHOS inhibition also promoted the transfer of mitochondria from cocultured mesenchymal stem cells, which rescued cells from apoptosis. To improve treatment, synergy with another ETC Complex I inhibitor, mubritinib, was explored (2). Alterations to other key metabolic pathways, such as fatty acid oxidation, are of interest when exploring AML chemotherapeutics. Similarly to IACS-010759, introduction of the short-chain fatty acid sodium propionate induced ROS, mitochondrial fission, and lethal PINK1- and ATG5-mediated mitophagy via ferroptosis and apoptosis (127). When AML mice were treated with sodium propionate, an anti-leukemic immune response was observed and overall survival increased (127).

Another group of compounds drawing attention for their ability to target mitochondria in AML are cannabinoids, particularly tetrahydrocannabinol (THC) and cannabidiol (CBD), which have been used commonly for palliative care (**Figure 2**). Each drug and extract has been shown to have antitumorigenic activity (128–130). Both cannabinoids bind to the cannabinoid receptors, CB1 and/or CB2, which are often expressed in AML cells (131). Binding of THC to CB receptors triggers apoptosis in both ALL and AML cell lines, but this response is thought to depend on lymphoid differentiation markers that vary amongst patient cohorts, suggesting that a personalized approach would be needed for this course of treatment to be viable (131). A similar outcome has been observed with CBD, which induces cell death in ALL and CML cell lines (132). Interestingly, CBD treatment appears to have pleiotropic, dose-dependent effects. High concentrations (>30 µM) trigger apoptosis, while lower concentrations (10 µM) activate autophagy. CBD also activates the PINK1-Parkin pathway (133). CBD has also been shown to disrupt the mitochondrial membrane potential, so this result is unsurprising (133). Considering that cyclosporin A reverses CBD-induced mitochondrial depolarization, Parkin recruitment is suggested to result from the formation and opening of the mitochondrial permeability transition pore (133). In CML, where CBD appears to cause similar effects on mitochondria and mitophagic activation, CBD showed synergy with the tyrosine kinase inhibitor, imatinib, the standard treatment for CML, including cytotoxic activity against imatinib-resistant cells (134).

Another mitophagic stimulator showing some promise in pre-clinical testing is the ceramide analog LCL-461 (**Figure 2**). This drug kills AML patient treatment-resistant blasts with an FLT3-ITD mutation (109). This mutation suppresses ceramide metabolism in AML patients, particularly the production of CerS1-generated C_18_-ceramides. C_18_-ceramide allows the execution of lethal mitophagy (109) and is currently thought to be incorporated into the outer mitochondrial membrane by an elegant dance (135). First, cell stress causes dissociation of p17/PERMIT from the mitochondrial fission protein Drp1, allowing the latter to trigger fission and freeing the former to find a new binding partner, in this case CerS1. This relocalizes CerS1 from the endoplasmic reticulum to mitochondria and facilitates C_18_-ceramide incorporation into the outer mitochondrial membrane by binding to MAP1-LC3 (135). This interaction signals for autophagosomes to execute lethal mitophagy (65, 109).

Treatment effectiveness for multi-kinase inhibitors with effects on FLT3 (e.g., sorafenib or crenolanib) depends on reactivation of C_18_-ceramide production. Understandably, reducing C_18_-ceramide synthesis by inhibiting CerS prevented these compounds from inducing AML cell death (109). Sorafenib was later found to inhibit both Complex II and Complex III of the mitochondrial ETC, disrupting mitochondrial membrane potential (136). This dual inhibition stabilizes PINK1 on the outer mitochondrial membrane, leading to mitophagic activation. It is also possible that the mechanism of action for the previously-mentioned C_6_-ceramide-mediated initiation of mitophagy operates through conversion of C_6_-ceramide to C_18_-ceramide (109), a conversion that is known to occur (137). Interestingly, mutation of ASXL1 in AML also causes low-levels of mitochondria-derived vesicles that are implicated in mitochondrial repair and mitophagy (120). This presents the intriguing possibility that LCL-461, or a similar ceramide analog, may also work as a novel anticancer treatment strategy in patients with this type of mutation as well.

Finally, hemin, a physiological erythroid maturation stimulator, was found to induce mitochondrial sequestration in enlarged autophagic vacuoles in the CML cell line K562 (138). Upon treatment with hemin, mitochondria were depolarized, although viability was not compromised. However, localization of the apoptotic protein Nix onto the mitochondrial membrane did trigger mitophagic activation (138). While this was observed in K562, the effect of hemin may translate to other leukemia models for therapeutic purposes and merits further investigation.

### 3.4 Mitophagic induction improves outcome when combined with standard clinical treatment

It has often been observed that single-target drugs lead to acquired resistance amongst AML cells (e.g., IDH1 treatments, FLT3 inhibitors, etc.) rendering them less effective or even completely useless. Remission is infrequent and resistance is commonplace. For this reason, AML treatment commonly utilizes drug combinations rather than monotherapies. One burgeoning idea is to combine existing chemotherapeutic treatments with mitophagic regulators. Although inhibiting mitophagy can stimulate cell death in AML, activating this pathway has been subject to more study (114, 139, 140). While it initially seems counterintuitive, excessive activation of mitophagy can reduce survival of leukemia cells (114, 139). Early results suggest that these combinations will be more effective and have reduced off-target effects.

For example, the combination of araC and mitoxantrone (a topoisomerase inhibitor) with devimistat, a CAC cycle inhibitor that induces mitochondrial ROS production, triggers mitophagy (141). Combining devimistat with chloroquine sensitizes AML blasts derived from patient samples. Interestingly, devimistat-induced mitophagy via ROS is independent of PINK1/Parkin (141). Another promising outcome from this study was that increased patient age correlated well with the level of mitochondrial ROS induced by devimistat, suggesting that this treatment may be effective in older patients who often have few treatment options (141). Indeed, mouse AML models with three different baselines of mitochondrial membrane potential showed different responses to devimistat, with a negative correlation between the two variables. Early trial of these drug combinations showed promising results, with a 50% rate of complete remission in patients with relapsed or refractory AML (142). A second study (NCT#02484391) has been conducted, but maintenance devimistat was unfortunately only administered in 2 of 21 responders (141). A third trial (NCT#03504410) complicated this analysis, as patients had poor outcomes and no significant difference between the devimistat treatment and control group were seen, leading to termination of the study (143). Unfortunately, devimistat-treated AML cells respond by increasing their dependency on gluconeogenesis, suggesting a mechanism of metabolic escape in AML that may limit treatment viability (141).

The novel PINK1 Stabilizing compounds discovered in our lab displayed promising synergy when combined with existing chemotherapeutics such as doxorubicin or 6-mercaptopurine in the treatment of AML cells (114). These strong synergistic interactions were highly specific to AML cells, as compared to healthy PBMCs, and effectively killed AML cells that were otherwise resistant to treatment. Interestingly, the synergy and selectivity of PINK1-stabilizing compound in combinations with standard AML treatments were higher than the combination of araC and doxorubicin under similar conditions. Treatment combinations of PS127 compounds with conventional chemotherapeutics were also effective against primary AML cells, although we observed interesting differences in effectiveness that appeared to correlate with AML subtypes (e.g., *de novo* vs. secondary AML, high or low percentage of blast cells, etc.) (114). While further development of these compounds is necessary, they show strong proof-of-concept for this approach.

### 3.5 Inhibiting autophagy while simultaneously inducing mitophagy reduced AML survival

Intriguingly, some evidence suggests that inducing lethal mitophagy can be even more effective if autophagy is simultaneously inhibited (84, 85). As has been seen in numerous other conditions, failure of proper autophagosomal formation during mitophagy shifts cells to an apoptotic fate (65, 144). For example, the pharmacological agents bafilomycin A1 (BafA1), chloroquine, and its derivative, Lys05, block autophagy by preventing lysosomal acidification (84, 85). Treatment with any of the three inhibitors increased mitochondrial content under hypoxic conditions (where mitophagy is activated) in AML cells, but not in normal hematopoietic cells. Unlike the other two drugs, BafA1 also exerted its effect in normoxia (84). BafA1 treatment reduced basal and maximal respiration in AML cells, consistent with previous findings that BafA1 induces OXPHOS uncoupling and mitochondrial depolarization (84, 145). ROS production and PINK1 stabilization were also observed in AML cells after treatment with BafA1 (**Figure 2**). This indicates that BafA1 disrupts mitochondrial function and induces the first steps of mitophagy while preventing completion of mitochondrial degradation. BafA1 treatment was also effective against LSCs. This promising combination also showed greater efficacy in reducing the *in vivo* tumor burden in mice engrafted with human AML or ALL cell lines (85, 146). Treating araC-resistant AML cells with BafA1 did not restore sensitivity to the chemotherapeutic (147). However, combined treatment of cells with BafA1 and commercial chemotherapeutics (arsenic trioxide (ATO), retinoic acid (ATRA), or araC) induced leukemic cell death, indicating a potential for therapeutic application (148, 149).

Similarly, combination of the hypomethylating agent OR21 with venetoclax significantly increased apoptotic induction through reduction of the mitochondrial homeostatic protein VAMP7, a SNARE family protein responsible for autophagosome formation (150). The combination of venetoclax and OR21 also significantly induced ROS production and mitochondrial damage (**Figure 2**). A xenografted mouse model treated with this combination showed significantly increased survival compared to single-drug treatment, without weight loss or severe toxicity (150).

## 4 Omics and functional assays to identify mitochondrial druggable proteins

As mentioned above, many AML treatments act by disrupting mitochondria to ultimately induce cell death, highlighting the value of probing the structure and function of this organelle for identifying additional chemotherapeutics. Potential drug targets may include mitochondrial translation, ROS production, apoptosis, and energy production (49, 83, 151). However, these are not ‘one size fits all’ therapeutic targets. For example, inducing ROS production can trigger autophagy-induced cell death, but it can also promote oxidative DNA damage favoring carcinogenesis or drug resistance (152–154).

Genetic and transcriptional profiling of AML cells has permitted AML classification based on their cytogenetic mutations and predicting leukemogenesis (155–157). Identifying patterns of co-mutations (whether gain or loss of function) is a useful tool for evaluating key mutations driving relapses (158). Using high-throughput sequencing technology, genetic mutations, gene expression, and epigenetic marking in leukemic cell lines, tumor samples and non-cancerous tissue samples were identified, published, and stored in The Cancer Genome Atlas and LeukemiaDB (159, 160). These databases significantly accelerated the development of diagnostic markers and targeted therapies.

Although transcriptional changes generally match genetic mutations, there are occasional differences, highlighting the importance of evaluating both in the study of cancers (161). Transcriptomics can shed light on gene expression levels and gene functions, which can be used to predict AML progression, relapse risk, and its response to treatment. For example, transcriptional profiling studies have shown that changes in expression of S100A8 and S100A9 alter sensitivity to venetoclax and gilteritinib (162, 163). This profile can also be used to predict overall survival as a function of LYPD3 expression (164). Nonetheless, patients with similar genetic mutations may respond differently to chemotherapy, as the effects of individual mutations are difficult to predict, in part due to other mutations and metabolic variations (165, 166).

The transcriptomes of drug-resistant lines have also been investigated to uncover genes responsible for drug resistance and are subsequently targeted to regain drug sensitivity. This was shown by the identification of mitochondrial translation genes whose disruption could restore sensitivity to venetoclax in otherwise resistant AML cells using a CRISPR screen (163, 167). Additionally, the Cap Analysis Gene Expression (CAGE) transcriptomic approach identified that various genes implemented in cell adhesion and actin polymerization were differentially regulated in OXPHOS-inhibitor resistant patient cells. CAGE also helped identify that mitochondrial fission followed by removal of damaged mitochondria by mitophagy facilitates resistance to OXPHOS inhibitors (67). Importantly, transcriptional profiling is likely to be a key contributor to the development of personalized treatments for AML, a long-sought goal that is beginning to come to fruition and is showing positive results in small clinical trials (168).

Unfortunately, research suggests that transcriptional profiling may also be insufficient for developing personalized medicine and that proteomic analysis could be necessary as well (169). In particular, proteomic profiling of bone marrow cells showed post-translational changes in protein abundance, despite the same levels of RNA transcripts (170). Furthermore, post-translation modifications affect protein functions required for cellular signaling and can further differentiate AML types. For example, changes to phosphorylation profoundly affect AML cell biology, and also need to be taken into account (168, 170–173). Treatment with an FLT3 inhibitor promotes phosphorylation of proteins related to autophagy and mTOR signaling, pinpointing the synergistic cytotoxicity of FLT3 and autophagic inhibitor (173). In attempts to find combinatorial treatments, proteomic and phosphoproteomic (protein phosphorylation profiles) features rationalize cell responses to co-treatments. Proteogenomics identified a specific mitochondrial protein pattern, termed C-Mito, that identifies hypersensitized AML cells to venetoclax and ETC Complex I inhibitor treatment (174). C-Mito provides additional classification of AML based on mitochondrial protein expression not reflected in the classical cellular differentiation markers. Notably, C-Mito-positive patients have a poorer overall probability of survival and worse remission rates than non-C-Mito patients (174).

Although several studies identified potential drug targets using single omics approaches, integrating multi-omics analyses and functional studies may be more promising for enabling a thorough understanding of AML pathogenesis and validate mechanisms of action of drugs. But these data may also enable the identification of actionable targets for treatment. For example, somatic mtDNA analysis of AML cells revealed mutations in genes encoding ETC Complex I, III, or IV (175). As previously discussed, many drugs inhibiting ETC examined in clinical trials, such as IACS-010759 and mubritinib (2, 151).

Transcriptomic, proteomic, and lipidomic approaches also identified a novel druggable target for AML that exploits the regulation of fatty acid oxidation by the mitochondrial deacetylase, Sirtuin-3 (SIRT3). Specifically, SIRT3 inhibition suppresses fatty acid oxidation, causing reduction of OXPHOS and ATP level, which leads to LSCs vulnerability to lipotoxicity (176). LSCs upregulate cholesterol metabolism to tolerate lipid accumulation causing cell death and lipotoxicity, suggesting potential mechanism to sensitize LSCs to SIRT3 inhibition.

## 5 Conclusion

The metabolic reprogramming required to support AML, favoring OXPHOS and fatty acid oxidation, precariously balances their mitochondrial activity, sensitizing them to any further mitochondrial disruption. This highlights the value of targeting mitochondria for therapeutic intervention. Although the dual role of mitochondrial metabolic pathways may currently pose a challenge for the effectiveness of current commercial treatments, they are the most likely route for moving forward in developing effective, long-term treatments for AML.

Targeting mitophagy has shown desirable effects in providing a new treatment modality and in abrogating resistance to conventional chemotherapeutics in AML cells. Tests of both mitophagic blockers (XRK3F2, S1g-2) and inducers (PS compounds, Hemin, CBD, LCL-461, devimistat) have shown positive effects in specifically targeting leukemic cells with minimal effects on healthy hematopoietic cells. These results, while initially surprising, suggest that either method can lead to the same end: damaged and dysfunctional mitochondria and apoptotic initiation. Clearly further investigation is needed to determine which course of treatment is most effective, but we anticipate that this research will demonstrate that optimized treatments will vary based on each patient’s genetic and metabolic state. For example, inducing mitophagy in AML has been most effective at destroying cancer cells that harbored FLT3-ITD mutations. It should be noted, however, that most of the studies testing this approach have used cells harboring this mutation. It is imperative that a wider panel of AML mutations be tested with combinations of mitophagic activators or inhibitors alone and with conventional chemotherapeutics, to evaluate the practicality of this therapeutic approach more generally.

## References

[1] Blum W Matthews V. Fast Facts: Acute myeloid leukemia. Abingdon, Oxford OX14 3LN, UK: Karger (2018).

[2] Baccelli I Gareau Y Lehnertz B Gingras S Spinella J-F Corneau S. Mubritinib targets the electron transport chain complex I and reveals the landscape of OXPHOS dependency in acute myeloid leukemia. Cancer Cell. (2019) 36:84–99.e8. doi: 10.1016/j.ccell.2019.06.003 31287994

[3] Panina SB Baran N Brasil Da Costa FH Konopleva M Kirienko NV. A mechanism for increased sensitivity of acute myeloid leukemia to mitotoxic drugs. Cell Death Dis. (2019) 10:617. doi: 10.1038/s41419-019-1851-3 31409768 PMC6692368

[4] Nelson MA McLaughlin KL Hagen JT Coalson HS Schmidt C Kassai M. Intrinsic OXPHOS limitations underlie cellular bioenergetics in leukemia. eLife. (2021) 10:e63104. doi: 10.7554/eLife.63104 34132194 PMC8221809

[5] De Beauchamp L Himonas E Helgason GV. Mitochondrial metabolism as a potential therapeutic target in myeloid leukaemia. Leukemia. (2022) 36:1–12. doi: 10.1038/s41375-021-01416-w 34561557 PMC8727299

[6] Xiao Y Hu B Guo Y Zhang D Zhao Y Chen Y. Targeting glutamine metabolism as an attractive therapeutic strategy for acute myeloid leukemia. Curr Treat Options Oncol. (2023) 24:1021–35. doi: 10.1007/s11864-023-01104-0 PMC10356674 37249801

[7] Hameed KM Bollino DR Shetty AC Carter-Cooper B Lapidus RG Emadi A. Dual targeting of glutamine and serine metabolism in acute myeloid leukemia. Front Oncol. (2024) 14:1326754. doi: 10.3389/fonc.2024.1326754 38690164 PMC11059989

[8] Frisch A Rowe JM Ofran Y. The increasingly blurred line between induction, consolidation and maintenance in acute myeloid leukaemia. Br J Haematol. (2023) 200:556–62. doi: 10.1111/bjh.18613 36572392

[9] Schlenk RF Weber D Fiedler W Salih HR Wulf G Salwender H. Midostaurin added to chemotherapy and continued single-agent maintenance therapy in acute myeloid leukemia with *FLT3*-ITD. Blood. (2019) 133:840–51. doi: 10.1182/blood-2018-08-869453 30563875

[10] Vetrie D Helgason GV Copland M. The leukaemia stem cell: similarities, differences and clinical prospects in CML and AML. Nat Rev Cancer. (2020) 20:158–73. doi: 10.1038/s41568-019-0230-9 31907378

[11] Loke J Buka R Craddock C. Allogeneic stem cell transplantation for acute myeloid leukemia: who, when, and how? Front Immunol. (2021) 12:659595. doi: 10.3389/fimmu.2021.659595 34012445 PMC8126705

[12] Chen T-T Ching-Chan L Lo W-J Hsieh C-Y Lein M-Y Che-Hung L. Venetoclax plus azacitidine as a bridge treatment to allogeneic stem cell transplantation in unfit patients with acute myeloid leukemia. Cancers. (2024) 16:1082. doi: 10.3390/cancers16061082 38539418 PMC10968407

[13] Willasch AM Peters C Sedláček P Dalle J-H Kitra-Roussou V Yesilipek A. Myeloablative conditioning for allo-HSCT in pediatric ALL: FTBI or chemotherapy?—A multicenter EBMT-PDWP study. Bone Marrow Transplant. (2020) 55:1540–51. doi: 10.1038/s41409-020-0854-0 PMC8376634 32203263

[14] Mao Y Yu C Hsieh T-S Nitiss JL Liu AA Wang H. Mutations of human topoisomerase IIα Affecting multidrug resistance and sensitivity. Biochemistry. (1999) 38:10793–800. doi: 10.1021/bi9909804 10451375

[15] Okada Y Tosaka A Nimura Y Kikuchi A Yoshida S Suzuki M. Atypical multidrug resistance may be associated with catalytically active mutants of human DNA topoisomerase II α. Gene. (2001) 272:141–8. doi: 10.1016/S0378-1119(01)00554-6 11470519

[16] Zhitomirsky B Assaraf YG. Lysosomes as mediators of drug resistance in cancer. Drug Resist Updat. (2016) 24:23–33. doi: 10.1016/j.drup.2015.11.004 26830313

[17] Niu J Peng D Liu L. Drug resistance mechanisms of acute myeloid leukemia stem cells. Front Oncol. (2022) 12:896426. doi: 10.3389/fonc.2022.896426 35865470 PMC9294245

[18] Stegmann AP Honders MW Kester MG Landegent JE Willemze R. Role of deoxycytidine kinase in an *in vitro* model for AraC- and DAC-resistance: substrate-enzyme interactions with deoxycytidine, 1-beta-D-arabinofuranosylcytosine and 5-aza-2’-deoxycytidine. Leukemia. (1993) 7:1005–11. 7686601

[19] Fanciullino R Farnault L Donnette M Imbs D-C Roche C Venton G. CDA as a predictive marker for life-threatening toxicities in patients with AML treated with cytarabine. Blood Adv. (2018) 2:462–9. doi: 10.1182/bloodadvances.2017014126 PMC5851420 29490977

[20] Wu B Mao ZJ Wang Z Wu P Huang H Zhao W. Deoxycytidine kinase (DCK) mutations in human acute myeloid leukemia resistant to cytarabine. Acta Haematol. (2021) 144:534–41. doi: 10.1159/000513696 33626530

[21] Li W Zhang H Assaraf YG Zhao K Xu X Xie J. Overcoming ABC transporter-mediated multidrug resistance: Molecular mechanisms and novel therapeutic drug strategies. Drug Resist Updat. (2016) 27:14–29. doi: 10.1016/j.drup.2016.05.001 27449595

[22] Long BH Wang L Lorico A Wang RC Brattain MG Casazza AM. Mechanisms of resistance to etoposide and teniposide in acquired resistant human colon and lung carcinoma cell lines. Cancer Res. (1991) 51:5275–83. 1717144

[23] Hazlehurst LA Foley NE Gleason-Guzman MC Hacker MP Cress AE Greenberger LW. Multiple mechanisms confer drug resistance to mitoxantrone in the human 8226 myeloma cell line. Cancer Res. (1999) 59:1021–8. 10070958

[24] Legrand O Zittoun R Marie J-P. Role of MRP1 in multidrug resistance in acute myeloid leukemia. Leuk. (1999) 13:578–84. doi: 10.1038/sj.leu.2401361 10214864

[25] Assaraf YG. The role of multidrug resistance efflux transporters in antifolate resistance and folate homeostasis. Drug Resist Updat. (2006) 9:227–46. doi: 10.1016/j.drup.2006.09.001 17092765

[26] Marcelletti JF Sikic BI Cripe LD Paietta E. Evidence of a role for functional heterogeneity in multidrug resistance transporters in clinical trials of P-glycoprotein modulation in acute myeloid leukemia. Cytometry B Clin Cytom. (2019) 96:57–66. doi: 10.1002/cyto.b.21737 30334334 PMC6340737

[27] Williams MS Amaral FM Simeoni F Somervaille TC. A stress-responsive enhancer induces dynamic drug resistance in acute myeloid leukemia. J Clin Invest. (2020) 130:1217. doi: 10.1172/JCI130809 31770110 PMC7269587

[28] Molica M Perrone S Mazzone C Niscola P Cesini L Abruzzese E. CD33 expression and gentuzumab ozogamicin in acute myeloid leukemia: two sides of the same coin. Cancers. (2021) 13:3214. doi: 10.3390/cancers13133214 34203180 PMC8268215

[29] Inase A Maimaitili Y Kimbara S Mizutani Y Miyata Y Ohata S. GSK3 inhibitor enhances gemtuzumab ozogamicin-induced apoptosis in primary human leukemia cells by overcoming multiple mechanisms of resistance. EJHaem. (2023) 4:153–64. doi: 10.1002/jha2.600 PMC9928658 36819180

[30] Chiara F Rasola A. GSK-3 and mitochondria in cancer cells. Front Oncol. (2013) 3:16. doi: 10.3389/fonc.2013.00016 23386998 PMC3564062

[31] Ye H Adane B Khan N Sullivan T Minhajuddin M Gasparetto M. Leukemic stem cells evade chemotherapy by metabolic adaptation to an adipose tissue niche. Cell Stem Cell. (2016) 19:23–37. doi: 10.1016/j.stem.2016.06.001 27374788 PMC4938766

[32] Jones CL Stevens BM D’Alessandro A Reisz JA Culp-Hill R Nemkov T. Inhibition of amino acid metabolism selectively targets human leukemia stem cells. Cancer Cell. (2018) 34:724–740.e4. doi: 10.1016/j.ccell.2018.10.005 30423294 PMC6280965

[33] Nwosu GO Ross DM Powell JA Pitson SM. Venetoclax therapy and emerging resistance mechanisms in acute myeloid leukaemia. Cell Death Dis. (2024) 15:1–11. doi: 10.1038/s41419-024-06810-7 38866760 PMC11169396

[34] Sriskanthadevan S Jeyaraju DV Chung TE Prabha S Xu W Skrtic M. AML cells have low spare reserve capacity in their respiratory chain that renders them susceptible to oxidative metabolic stress. Blood. (2015) 125:2120–30. doi: 10.1182/blood-2014-08-594408 PMC4375109 25631767

[35] Farge T Saland E De Toni F Aroua N Hosseini M Perry R. Chemotherapy-resistant human acute myeloid leukemia cells are not enriched for leukemic stem cells but require oxidative metabolism. Cancer Discovery. (2017) 7:716–35. doi: 10.1158/2159-8290.CD-16-0441 PMC5501738 28416471

[36] Panina SB Pei J Kirienko NV. Mitochondrial metabolism as a target for acute myeloid leukemia treatment. Cancer Metab. (2021) 9:17. doi: 10.1186/s40170-021-00253-w 33883040 PMC8058979

[37] Samudio I Harmancey R Fiegl M Kantarjian H Konopleva M Korchin B. Pharmacologic inhibition of fatty acid oxidation sensitizes human leukemia cells to apoptosis induction. J Clin Invest. (2010) 120:142–56. doi: 10.1172/JCI38942 PMC2799198 20038799

[38] DiNardo CD Cortes JE. Mutations in AML: prognostic and therapeutic implications. Hematol Am Soc Hematol Educ Program. (2016) 2016:348–55. doi: 10.1182/asheducation-2016.1.348 PMC6142505 27913501

[39] Liberti MV Locasale JW. The warburg effect: how does it benefit cancer cells? Trends Biochem Sci. (2016) 41:211–8. doi: 10.1016/j.tibs.2015.12.001 PMC4783224 26778478

[40] Jalte M Abbassi M El Mouhi H Daha Belghiti H Ahakoud M Bekkari H. FLT3 mutations in acute myeloid leukemia: unraveling the molecular mechanisms and implications for targeted therapies. Cureus. (2023) 15:e45765. doi: 10.7759/cureus.45765 37872917 PMC10590537

[41] Alshamleh I Kurrle N Makowka P Bhayadia R Kumar R Süsser S. PDP1 is a key metabolic gatekeeper and modulator of drug resistance in FLT3-ITD-positive acute myeloid leukemia. Leukemia. (2023) 37:2367–82. doi: 10.1038/s41375-023-02041-5 PMC10681906 37935978

[42] Kiyoi H Kawashima N Ishikawa Y. FLT3 mutations in acute myeloid leukemia: Therapeutic paradigm beyond inhibitor development. Cancer Sci. (2020) 111:312–22. doi: 10.1111/cas.14274 PMC7004512 31821677

[43] Short NJ Nguyen D Ravandi F. Treatment of older adults with FLT3-mutated AML: Emerging paradigms and the role of frontline FLT3 inhibitors. Blood Cancer J. (2023) 13:142. doi: 10.1038/s41408-023-00911-w 37696819 PMC10495326

[44] Antar AI Otrock ZK Jabbour E Mohty M Bazarbachi A. FLT3 inhibitors in acute myeloid leukemia: ten frequently asked questions. Leukemia. (2020) 34:682–96. doi: 10.1038/s41375-019-0694-3 31919472

[45] Larrosa-Garcia M Baer MR. FLT3 inhibitors in acute myeloid leukemia: Current status and future directions. Mol Cancer Ther. (2017) 16:991. doi: 10.1158/1535-7163.MCT-16-0876 28576946 PMC5600895

[46] Stone RM Mandrekar SJ Sanford BL Laumann K Geyer S Bloomfield CD. Midostaurin plus chemotherapy for acute myeloid leukemia with a FLT3 mutation. N Engl J Med. (2017) 377:454–64. doi: 10.1056/NEJMoa1614359 PMC5754190 28644114

[47] Zhao JC Agarwal S Ahmad H Amin K Bewersdorf JP Zeidan AM. A review of FLT3 inhibitors in acute myeloid leukemia. Blood Rev. (2022) 52:100905. doi: 10.1016/j.blre.2021.100905 34774343 PMC9846716

[48] Sierra J Montesinos P Thomas X Griskevicius L Cluzeau T Caillot D. Midostaurin plus daunorubicin or idarubicin for young and older adults with FLT3-mutated AML: a phase 3b trial. Blood Adv. (2023) 7:6441. doi: 10.1182/bloodadvances.2023009847 37581981 PMC10632658

[49] Chen X Glytsou C Zhou H Narang S Reyna DE Lopez A. Targeting mitochondrial structure sensitizes acute myeloid leukemia to venetoclax treatment. Cancer Discovery. (2019) 9:890–909. doi: 10.1158/2159-8290.CD-19-0117 31048321 PMC6606342

[50] Burchert A Bug G Fritz LV Finke J Stelljes M Röllig C. Sorafenib maintenance after allogeneic hematopoietic stem cell transplantation for acute myeloid leukemia with *FLT3* –internal tandem duplication mutation (SORMAIN). J Clin Oncol. (2020) 38:2993–3002. doi: 10.1200/JCO.19.03345 32673171

[51] Griffin JD Song Y Yang H Freimark J Shah MV. Post-transplant maintenance therapy in patients with FLT3-mutated acute myeloid leukemia: Real-world treatment patterns and outcomes. Eur J Haematol. (2021) 107:553. doi: 10.1111/ejh.13692 34289175 PMC9292256

[52] Aiman W Ali MA Basit MA Omar Z Suleman M Hassan M. Efficacy and tolerability of isocitrate dehydrogenase inhibitors in patients with acute myeloid leukemia: A systematic review of clinical trials. Leuk Res. (2023) 129:107077. doi: 10.1016/j.leukres.2023.107077 37100025

[53] Chen X Xing H Xie X Kou L Li J Li Y. Efficacy and safety of FDA-approved IDH inhibitors in the treatment of IDH mutated acute myeloid leukemia: a systematic review and meta-analysis. Clin Epigenetics. (2023) 15:113. doi: 10.1186/s13148-023-01529-2 37434249 PMC10334617

[54] Stuani L Sabatier M Saland E Cognet G Poupin N Bosc C. Mitochondrial metabolism supports resistance to IDH mutant inhibitors in acute myeloid leukemia. J Exp Med. (2021) 218:e20200924. doi: 10.1084/jem.20200924 33760042 PMC7995203

[55] Perl AE Altman JK Cortes J Smith C Litzow M Baer MR. Selective inhibition of FLT3 by gilteritinib in relapsed/refractory acute myeloid leukemia: a multicenter, first-in-human, open-label, phase 1/2 study. Lancet Oncol. (2017) 18:1061. doi: 10.1016/S1470-2045(17)30416-3 28645776 PMC5572576

[56] Intlekofer AM Shih AH Wang B Nazir A Rustenburg AS Albanese SK. Acquired resistance to IDH inhibition through trans or cis dimer-interface mutations. Nature. (2018) 559:125. doi: 10.1038/s41586-018-0251-7 29950729 PMC6121718

[57] Liu J Chen Y Yu L Yang L. Mechanisms of venetoclax resistance and solutions. Front Oncol. (2022) 12:1005659. doi: 10.3389/fonc.2022.1005659 36313732 PMC9597307

[58] Catalano G Zaza A Banella C Pelosi E Castelli G de Marinis E. MCL1 regulates AML cells metabolism via direct interaction with HK2. Metabolic signature at onset predicts overall survival in AMLs’ patients. Leukemia. (2023) 37:1600–10. doi: 10.1038/s41375-023-01946-5 37349598

[59] Divakaruni AS Brand MD. The regulation and physiology of mitochondrial proton leak. Physiology. (2011) 26:192–205. doi: 10.1152/physiol.00046.2010 21670165

[60] Zhao R-Z Jiang S Zhang L Yu Z-B. Mitochondrial electron transport chain, ROS generation and uncoupling (Review). Int J Mol Med. (2019) 44:3–15. doi: 10.3892/ijmm.2019.4188 31115493 PMC6559295

[61] Romo-González M Ijurko C Hernández-Hernández Á. Reactive oxygen species and metabolism in leukemia: A dangerous liaison. Front Immunol. (2022) 13:889875. doi: 10.3389/fimmu.2022.889875 35757686 PMC9218220

[62] Tcheng M Roma A Ahmed N Smith RW Jayanth P Minden MD. Very long chain fatty acid metabolism is required in acute myeloid leukemia. Blood. (2021) 137:3518–32. doi: 10.1182/blood.2020008551 PMC8225921 33720355

[63] Kao L-P Morad SAF Davis TS MacDougall MR Kassai M Abdelmageed N. Chemotherapy selection pressure alters sphingolipid composition and mitochondrial bioenergetics in resistant HL-60 cells. J Lipid Res. (2019) 60:1590–602. doi: 10.1194/jlr.RA119000251 PMC6718434 31363040

[64] Liu Y-Y Yu JY Yin D Patwardhan GA Gupta V Hirabayashi Y. A role for ceramide in driving cancer cell resistance to doxorubicin. FASEB J Off Publ Fed Am Soc Exp Biol. (2008) 22:2541–51. doi: 10.1096/fj.07-092981 18245173

[65] Sentelle RD Senkal CE Jiang W Ponnusamy S Gencer S Panneer Selvam S. Ceramide targets autophagosomes to mitochondria and induces lethal mitophagy. Nat Chem Biol. (2012) 8:831–8. doi: 10.1038/nchembio.1059 PMC3689583 22922758

[66] Marlein CR Zaitseva L Piddock RE Robinson SD Edwards DR Shafat MS. NADPH oxidase-2 derived superoxide drives mitochondrial transfer from bone marrow stromal cells to leukemic blasts. Blood. (2017) 130:1649–60. doi: 10.1182/blood-2017-03-772939 28733324

[67] Saito K Zhang Q Yang H Yamatani K Ai T Ruvolo V. Exogenous mitochondrial transfer and endogenous mitochondrial fission facilitate AML resistance to OxPhos inhibition. Blood Adv. (2021) 5:4233–55. doi: 10.1182/bloodadvances.2020003661 PMC8945617 34507353

[68] Zhao Z Mei Y Wang Z He W. The effect of oxidative phosphorylation on cancer drug resistance. Cancers. (2022) 15:62. doi: 10.3390/cancers15010062 36612059 PMC9817696

[69] Wise DR Thompson CB. Glutamine addiction: A new therapeutic target in cancer. Trends Biochem Sci. (2010) 35:427. doi: 10.1016/j.tibs.2010.05.003 20570523 PMC2917518

[70] Willems L Jacque N Jacquel A Neveux N Maciel TT Lambert M. Inhibiting glutamine uptake represents an attractive new strategy for treating acute myeloid leukemia. Blood. (2013) 122:3521. doi: 10.1182/blood-2013-03-493163 24014241 PMC3829119

[71] Emadi A Jun SA Tsukamoto T Fathi AT Minden MD Dang CV. Inhibition of glutaminase selectively suppresses the growth of primary acute myeloid leukemia cells with *IDH* mutations. Exp Hematol. (2014) 42:247–51. doi: 10.1016/j.exphem.2013.12.001 24333121

[72] Gregory MA Nemkov T Park HJ Zaberezhnyy V Gehrke S Adane B. Targeting glutamine metabolism and redox state for leukemia therapy. Clin Cancer Res. (2019) 25:4079–90. doi: 10.1158/1078-0432.CCR-18-3223 PMC6642698 30940653

[73] Kalyanaraman B Cheng G Hardy M Ouari O Lopez M Joseph J. A review of the basics of mitochondrial bioenergetics, metabolism, and related signaling pathways in cancer cells: Therapeutic targeting of tumor mitochondria with lipophilic cationic compounds. Redox Biol. (2018) 14:316–27. doi: 10.1016/j.redox.2017.09.020 PMC5633086 29017115

[74] Stueland CS Gorden K LaPorte DC. The isocitrate dehydrogenase phosphorylation cycle. Identification of the primary rate-limiting step. J Biol Chem. (1988) 263:19475–9. doi: 10.1016/S0021-9258(19)77658-3 3058700

[75] Song K Li M Xu X Xuan L Huang G Liu Q. Resistance to chemotherapy is associated with altered glucose metabolism in acute myeloid leukemia. Oncol Lett. (2016) 12:334. doi: 10.3892/ol.2016.4600 27347147 PMC4906727

[76] Roma A Tcheng M Ahmed N Walker S Jayanth P Minden MD. Glutamine metabolism mediates sensitivity to respiratory complex II inhibition in acute myeloid leukemia. Mol Cancer Res. (2022) 20:1659–73. doi: 10.1158/1541-7786.MCR-21-1032 35994381

[77] Ma G Zhang Z Li P Zhang Z Zeng M Liang Z. Reprogramming of glutamine metabolism and its impact on immune response in the tumor microenvironment. Cell Commun Signal. (2022) 20:114. doi: 10.1186/s12964-022-00909-0 35897036 PMC9327201

[78] Aquilano K Baldelli S Ciriolo MR. Glutathione: new roles in redox signaling for an old antioxidant. Front Pharmacol. (2014) 5:196. doi: 10.3389/fphar.2014.00196 25206336 PMC4144092

[79] Pei S Minhajuddin M Callahan KP Balys M Ashton JM Neering SJ. Targeting aberrant glutathione metabolism to eradicate human acute myelogenous leukemia cells. J Biol Chem. (2013) 288:33542–58. doi: 10.1074/jbc.M113.511170 PMC3837103 24089526

[80] Sillar JR Germon ZP DeIuliis GN Dun MD. The role of reactive oxygen species in acute myeloid leukaemia. Int J Mol Sci. (2019) 20:6003. doi: 10.3390/ijms20236003 31795243 PMC6929020

[81] Zhang J Gu Y Chen B. Mechanisms of drug resistance in acute myeloid leukemia. OncoTargets Ther. (2019) 12:1937–45. doi: 10.2147/OTT.S191621 PMC6417008 30881045

[82] Mesbahi Y Trahair TN Lock RB Connerty P. Exploring the metabolic landscape of AML: from haematopoietic stem cells to myeloblasts and leukaemic stem cells. Front Oncol. (2022) 12:807266. doi: 10.3389/fonc.2022.807266 35223487 PMC8867093

[83] Skrtić M Sriskanthadevan S Jhas B Gebbia M Wang X Wang Z. Inhibition of mitochondrial translation as a therapeutic strategy for human acute myeloid leukemia. Cancer Cell. (2011) 20:674–88. doi: 10.1016/j.ccr.2011.10.015 PMC3221282 22094260

[84] Fay HRS Dykstra KM Johnson M Cronin TL Lutgen-Dunckley L Martens BL. Mitophagy plays a key role in the anti-leukemic activity of autophagy inhibitors under hypoxia in acute myeloid leukemia. Blood. (2019) 134:1278–8. doi: 10.1182/blood-2019-127024

[85] Dykstra KM Fay HRS Massey AC Yang N Johnson M Portwood S. Inhibiting autophagy targets human leukemic stem cells and hypoxic AML blasts by disrupting mitochondrial homeostasis. Blood Adv. (2021) 5:2087–100. doi: 10.1182/bloodadvances.2020002666 PMC8095145 33877295

[86] Pei S Minhajuddin M Adane B Khan N Stevens BM Mack SC. AMPK/FIS1-mediated mitophagy is required for self-renewal of human AML stem cells. Cell Stem Cell. (2018) 23:86–100.e6. doi: 10.1016/j.stem.2018.05.021 29910151 PMC6035102

[87] Moschoi R Imbert V Nebout M Chiche J Mary D Prebet T. Protective mitochondrial transfer from bone marrow stromal cells to acute myeloid leukemic cells during chemotherapy. Blood. (2016) 128:253–64. doi: 10.1182/blood-2015-07-655860 27257182

[88] Forte D García-Fernández M Sánchez-Aguilera A Stavropoulou V Fielding C Martín-Pérez D. Bone marrow mesenchymal stem cells support acute myeloid leukemia bioenergetics and enhance antioxidant defense and escape from chemotherapy. Cell Metab. (2020) 32:829–843.e9. doi: 10.1016/j.cmet.2020.09.001 32966766 PMC7658808

[89] Schimmer AD. Mitochondrial shapeshifting impacts AML stemness and differentiation. Cell Stem Cell. (2018) 23:3–4. doi: 10.1016/j.stem.2018.05.026 29979990

[90] Lum JJ DeBerardinis RJ Thompson CB. Autophagy in metazoans: cell survival in the land of plenty. Nat Rev Mol Cell Biol. (2005) 6:439–48. doi: 10.1038/nrm1660 15928708

[91] Zhang S-P Niu Y-N Yuan N Zhang A-H Chao D Xu Q-P. Role of autophagy in acute myeloid leukemia therapy. Chin J Cancer. (2013) 32:130–5. doi: 10.5732/cjc.012.10073 PMC3845596 22854065

[92] Altman BJ Wofford JA Zhao Y Coloff JL Ferguson EC Wieman HL. Autophagy provides nutrients but can lead to chop-dependent induction of bim to sensitize growth factor–deprived cells to apoptosis. Mol Biol Cell. (2009) 20:1180–91. doi: 10.1091/mbc.E08-08-0829 PMC2642748 19109422

[93] Singh R Kaushik S Wang Y Xiang Y Novak I Komatsu M. Autophagy regulates lipid metabolism. Nature. (2009) 458:1131–5. doi: 10.1038/nature07976 PMC2676208 19339967

[94] Pizzino G Irrera N Cucinotta M Pallio G Mannino F Arcoraci V. Oxidative stress: harms and benefits for human health. Oxid Med Cell Longev. (2017) 2017:8416763. doi: 10.1155/2017/8416763 28819546 PMC5551541

[95] Cheng H Yang B Ke T Li S Yang X Aschner M. Mechanisms of metal-induced mitochondrial dysfunction in neurological disorders. Toxics. (2021) 9:142. doi: 10.3390/toxics9060142 34204190 PMC8235163

[96] Kowalczyk P Sulejczak D Kleczkowska P Bukowska-Ośko I Kucia M Popiel M. Mitochondrial oxidative stress—A causative factor and therapeutic target in many diseases. Int J Mol Sci. (2021) 22:13384. doi: 10.3390/ijms222413384 34948180 PMC8707347

[97] Reddam A McLarnan S Kupsco A. Environmental chemical exposures and mitochondrial dysfunction: a review of recent literature. Curr Environ Health Rep. (2022) 9:631. doi: 10.1007/s40572-022-00371-7 35902457 PMC9729331

[98] Nadalutti CA Ayala-Peña S Santos JH. Mitochondrial DNA damage as driver of cellular outcomes. Am J Physiol - Cell Physiol. (2021) 322:C136. doi: 10.1152/ajpcell.00389.2021 34936503 PMC8799395

[99] Liao S Chen L Song Z He H. The fate of damaged mitochondrial DNA in the cell. Biochim Biophys Acta BBA - Mol Cell Res. (2022) 1869:119233. doi: 10.1016/j.bbamcr.2022.119233 35131372

[100] Nguyen TD Shaid S Vakhrusheva O KosChade SE Klann K Thölken M. Loss of the selective autophagy receptor p62 impairs murine myeloid leukemia progression and mitophagy. Blood. (2019) 133:168–79. doi: 10.1182/blood-2018-02-833475 30498063

[101] Song C Pan S Zhang J Li N Geng Q. Mitophagy: A novel perspective for insighting into cancer and cancer treatment. Cell Prolif. (2022) 55:e13327. doi: 10.1111/cpr.13327 36200262 PMC9715364

[102] Yamano K Youle RJ. PINK1 is degraded through the N-end rule pathway. Autophagy. (2013) 9:1758–69. doi: 10.4161/auto.24633 PMC4028335 24121706

[103] Mouton-Liger F Jacoupy M Corvol J-C Corti O. PINK1/parkin-dependent mitochondrial surveillance: from pleiotropy to parkinson’s disease. Front Mol Neurosci. (2017) 10:120. doi: 10.3389/fnmol.2017.00120 28507507 PMC5410576

[104] Pickrell AM Youle RJ. The roles of PINK1, parkin, and mitochondrial fidelity in parkinson’s disease. Neuron. (2015) 85:257–73. doi: 10.1016/j.neuron.2014.12.007 PMC4764997 25611507

[105] Yamada T Dawson TM Yanagawa T Iijima M Sesaki H. SQSTM1/p62 promotes mitochondrial ubiquitination independently of PINK1 and PRKN/parkin in mitophagy. Autophagy. (2019) 15:2012–8. doi: 10.1080/15548627.2019.1643185 PMC6844492 31339428

[106] Chu CT Ji J Dagda RK Jiang JF Tyurina YY Kapralov AA. Cardiolipin externalization to the outer mitochondrial membrane acts as an elimination signal for mitophagy in neuronal cells. Nat Cell Biol. (2013) 15:1197–205. doi: 10.1038/ncb2837 PMC3806088 24036476

[107] Li X-X Tsoi B Li Y-F Kurihara H He R-R. Cardiolipin and its different properties in mitophagy and apoptosis. J Histochem Cytochem. (2015) 63:301–11. doi: 10.1369/0022155415574818 PMC4409943 25673287

[108] Ahmadpour ST Mahéo K Servais S Brisson L Dumas J-F. Cardiolipin, the mitochondrial signature lipid: implication in cancer. Int J Mol Sci. (2020) 21:8031. doi: 10.3390/ijms21218031 33126604 PMC7662448

[109] Dany M Gencer S Nganga R Thomas RJ Oleinik N Baron KD. Targeting FLT3-ITD signaling mediates ceramide-dependent mitophagy and attenuates drug resistance in AML. Blood. (2016) 128:1944–58. doi: 10.1182/blood-2016-04-708750 PMC5064718 27540013

[110] Meyer LM KosChade SE Vischedyk JB Thoelken M Gubas A Wegner M. Deciphering the mitophagy receptor network identifies a crucial role for OPTN (optineurin) in acute myeloid leukemia. Autophagy. (2023) 19:2982–96. doi: 10.1080/15548627.2023.2230839 PMC10549194 37439113

[111] Joffre C Ducau C Poillet-Perez L Courdy C Mansat-De Mas V. Autophagy a close relative of AML biology. Biology. (2021) 10:552. doi: 10.3390/biology10060552 34207482 PMC8235674

[112] Rodrigo R Mendis N Ibrahim M Ma C Kreinin E Roma A. Knockdown of BNIP3L or SQSTM1 alters cellular response to mitochondria target drugs. Autophagy. (2019) 15:900–7. doi: 10.1080/15548627.2018.1558002 PMC6526872 30563411

[113] Li Y Zheng W Lu Y Zheng Y Pan L Wu X. BNIP3L/NIX-mediated mitophagy: molecular mechanisms and implications for human disease. Cell Death Dis. (2021) 13:1–11. doi: 10.1038/s41419-021-04469-y 34930907 PMC8688453

[114] Panina SB Pei J Baran N Tjahjono E Patel S Alatrash G. Novel mitochondria-targeting compounds selectively kill human leukemia cells. Leukemia. (2022) 36:2009–21. doi: 10.1038/s41375-022-01614-0 PMC11088873 35672446

[115] Liu T Zhang L Joo D Sun S-C. NF-κB signaling in inflammation. Signal Transduct Target Ther. (2017) 2:1–9. doi: 10.1038/sigtrans.2017.23 PMC5661633 29158945

[116] Sanchez-Lopez E Ghia EM Antonucci L Sharma N Rassenti LZ Xu J. NF-κB-p62-NRF2 survival signaling is associated with high ROR1 expression in chronic lymphocytic leukemia. Cell Death Differ. (2020) 27:2206–16. doi: 10.1038/s41418-020-0496-1 PMC7308363 31992855

[117] Nepstad I Hatfield KJ Grønningsæter IS Reikvam H. The PI3K-akt-mTOR signaling pathway in human acute myeloid leukemia (AML) cells. Int J Mol Sci. (2020) 21:2907. doi: 10.3390/ijms21082907 32326335 PMC7215987

[118] Saxton RA Sabatini DM. mTOR signaling in growth, metabolism, and disease. Cell. (2017) 168:960–76. doi: 10.1016/j.cell.2017.02.004 PMC5394987 28283069

[119] Gumeni S Papanagnou E-D Manola MS Trougakos IP. Nrf2 activation induces mitophagy and reverses Parkin/Pink1 knock down-mediated neuronal and muscle degeneration phenotypes. Cell Death Dis. (2021) 12:1–12. doi: 10.1038/s41419-021-03952-w 34218254 PMC8254809

[120] Mondet J Lo Presti C Chevalier S Bertrand A Tondeur S Blanchet S. Mitochondria in human acute myeloid leukemia cell lines have ultrastructural alterations linked to deregulation of their respiratory profiles. Exp Hematol. (2021) 98:53–62.e3. doi: 10.1016/j.exphem.2021.03.001 33689800

[121] Oshima Y Cartier E Boyman L Verhoeven N Polster BM Huang W. Parkin-independent mitophagy via Drp1-mediated outer membrane severing and inner membrane ubiquitination. J Cell Biol. (2021) 220:e202006043. doi: 10.1083/jcb.202006043 33851959 PMC8050842

[122] Cho HM Ryu JR Jo Y Seo TW Choi YN Kim JH. Drp1-zip1 interaction regulates mitochondrial quality surveillance system. Mol Cell. (2019) 73:364–376.e8. doi: 10.1016/j.molcel.2018.11.009 30581142

[123] Li Y Li Y Yin J Wang C Yang M Gu J. A mitophagy inhibitor targeting p62 attenuates the leukemia-initiation potential of acute myeloid leukemia cells. Cancer Lett. (2021) 510:24–36. doi: 10.1016/j.canlet.2021.04.003 33862150

[124] Song T Yin F Wang Z Zhang H Liu P Guo Y. Hsp70-Bim interaction facilitates mitophagy by recruiting parkin and TOMM20 into a complex. Cell Mol Biol Lett. (2023) 28:46. doi: 10.1186/s11658-023-00458-5 37237369 PMC10223935

[125] Song T Guo Y Xue Z Guo Z Wang Z Lin D. Small-molecule inhibitor targeting the Hsp70-Bim protein–protein interaction in CML cells overcomes BCR-ABL-independent TKI resistance. Leukemia. (2021) 35:2862–74. doi: 10.1038/s41375-021-01283-5 34007045

[126] Tjahjono E Pei J Revtovich AV Liu T-JE Swadi A Hancu MC. Mitochondria-affecting small molecules ameliorate proteostasis defects associated with neurodegenerative diseases. Sci Rep. (2021) 11:17733. doi: 10.1038/s41598-021-97148-z 34489512 PMC8421394

[127] Wei Y Liu W Wang R Chen Y Liu J Guo X. Propionate promotes ferroptosis and apoptosis through mitophagy and ACSL4-mediated ferroptosis elicits anti-leukemia immunity. Free Radic Biol Med. (2024) 213:36–51. doi: 10.1016/j.freeradbiomed.2024.01.005 38215892

[128] Casanova ML Blázquez C Martínez-Palacio J Villanueva C Fernández-Aceñero MJ Huffman JW. Inhibition of skin tumor growth and angiogenesis *in vivo* by activation of cannabinoid receptors. J Clin Invest. (2003) 111:43–50. doi: 10.1172/JCI16116 12511587 PMC151833

[129] Lteif A Shebaby W El Hage M Azar-Atallah S Mroue D Mroueh M. Lebanese cannabis oil as a potential treatment for acute myeloid leukemia: *In vitro* and *in vivo* evaluations. J Ethnopharmacol. (2024) 333:118512. doi: 10.1016/j.jep.2024.118512 38964627

[130] Seltzer ES Watters AK MacKenzie D Granat LM Zhang D. Cannabidiol (CBD) as a promising anti-cancer drug. Cancers. (2020) 12:3203. doi: 10.3390/cancers12113203 33143283 PMC7693730

[131] Kampa-Schittenhelm KM Salitzky O Akmut F Illing B Kanz L Salih HR. Dronabinol has preferential antileukemic activity in acute lymphoblastic and myeloid leukemia with lymphoid differentiation patterns. BMC Cancer. (2016) 16:25. doi: 10.1186/s12885-015-2029-8 26775260 PMC4715874

[132] Olivas-Aguirre M Torres-López L Valle-Reyes JS Hernández-Cruz A Pottosin I Dobrovinskaya O. Cannabidiol directly targets mitochondria and disturbs calcium homeostasis in acute lymphoblastic leukemia. Cell Death Dis. (2019) 10:779. doi: 10.1038/s41419-019-2024-0 31611561 PMC6791884

[133] Ramirez A Old W Selwood DL Liu X. Cannabidiol activates PINK1-Parkin-dependent mitophagy and mitochondrial-derived vesicles. Eur J Cell Biol. (2022) 101:151185. doi: 10.1016/j.ejcb.2021.151185 34915361 PMC8816654

[134] Maggi F Morelli MB Tomassoni D Marinelli O Aguzzi C Zeppa L. The effects of cannabidiol via TRPV2 channel in chronic myeloid leukemia cells and its combination with imatinib. Cancer Sci. (2022) 113:1235–49. doi: 10.1111/cas.15257 PMC8990867 34971020

[135] Oleinik N Kim J Roth BM Selvam SP Gooz M Johnson RH. Mitochondrial protein import is regulated by p17/PERMIT to mediate lipid metabolism and cellular stress. Sci Adv. (2019) 5:eaax1978. doi: 10.1126/sciadv.aax1978 31535025 PMC6739097

[136] Zhang C Liu Z Bunker E Ramirez A Lee S Peng Y. Sorafenib targets the mitochondrial electron transport chain complexes and ATP synthase to activate the PINK1–Parkin pathway and modulate cellular drug response. J Biol Chem. (2017) 292:15105–20. doi: 10.1074/jbc.M117.783175 PMC5592685 28673964

[137] Morad SAF MacDougall MR Abdelmageed N Kao L-P Feith DJ Tan S-F. Pivotal role of mitophagy in response of acute myelogenous leukemia to a ceramide-tamoxifen-containing drug regimen. Exp Cell Res. (2019) 381:256–64. doi: 10.1016/j.yexcr.2019.05.021 PMC6909922 31112736

[138] Fader CM Salassa BN Grosso RA Vergara AN Colombo MI. Hemin induces mitophagy in a leukemic erythroblast cell line. Biol Cell. (2016) 108:77–95. doi: 10.1111/boc.201500058 26773440

[139] Kim EH Sohn S Kwon HJ Kim SU Kim M-J Lee S-J. Sodium selenite induces superoxide-mediated mitochondrial damage and subsequent autophagic cell death in Malignant glioma cells. Cancer Res. (2007) 67:6314–24. doi: 10.1158/0008-5472.CAN-06-4217 17616690

[140] Yao N Wang C Hu N Li Y Liu M Lei Y. Inhibition of PINK1/Parkin-dependent mitophagy sensitizes multidrug-resistant cancer cells to B5G1, a new betulinic acid analog. Cell Death Dis. (2019) 10:1–16. doi: 10.1038/s41419-019-1470-z PMC6408511 30850585

[141] Anderson R Miller LD Isom S Chou JW Pladna KM Schramm NJ. Phase II trial of cytarabine and mitoxantrone with devimistat in acute myeloid leukemia. Nat Commun. (2022) 13:1673. doi: 10.1038/s41467-022-29039-4 35354808 PMC8967916

[142] Pardee TS Anderson RG Pladna KM Isom S Ghiraldeli LP Miller LD. A phase I study of CPI-613 in combination with high-dose cytarabine and mitoxantrone for relapsed or refractory acute myeloid leukemia. Clin Cancer Res. (2018) 24:2060–73. doi: 10.1158/1078-0432.CCR-17-2282 PMC5932089 29437791

[143] Pardee TS Powell BL Larson RA Maly J Keng M Foster M. Devimistat plus chemotherapy vs chemotherapy alone for older relapsed or refractory patients with AML: results of the ARMADA trial. Blood Neoplasia. (2024) 1:100009. doi: 10.1016/j.bneo.2024.100009 PMC12082102 40454396

[144] Wang H Ye J Peng Y Ma W Chen H Sun H. CKLF induces microglial activation via triggering defective mitophagy and mitochondrial dysfunction. Autophagy. (2023) 20:590. doi: 10.1080/15548627.2023.2276639 37908119 PMC10936627

[145] Zhdanov AV Dmitriev RI Papkovsky DB. Bafilomycin A1 activates respiration of neuronal cells via uncoupling associated with flickering depolarization of mitochondria. Cell Mol Life Sci CMLS. (2010) 68:903. doi: 10.1007/s00018-010-0502-8 20820851 PMC3037485

[146] Yuan N Song L Zhang S Lin W Cao Y Xu F. Bafilomycin A1 targets both autophagy and apoptosis pathways in pediatric B-cell acute lymphoblastic leukemia. Haematologica. (2015) 100:345. doi: 10.3324/haematol.2014.113324 25512644 PMC4349273

[147] Visser N Lourens HJ Huls G Bremer E Wiersma VR. Inhibition of autophagy does not re-sensitize acute myeloid leukemia cells resistant to cytarabine. Int J Mol Sci. (2021) 22:2337. doi: 10.3390/ijms22052337 33652766 PMC7956277

[148] Nourkeyhani H Jason DHP Scott P Hanekamp D Johnson M Wang ES. Targeting autophagy as a therapeutic strategy in acute myeloid leukemia. Blood. (2016) 128:3950. doi: 10.1182/blood.V128.22.3950.3950

[149] Haghi A Salemi M Fakhimahmadi A Mohammadi Kian M Yousefi H Rahmati M. Effects of different autophagy inhibitors on sensitizing KG-1 and HL-60 leukemia cells to chemotherapy. IUBMB Life. (2021) 73:130–45. doi: 10.1002/iub.2411 33205598

[150] Kamachi K Ureshino H Watanabe T Yoshida-Sakai N Fukuda-Kurahashi Y Kawasoe K. Combination of a new oral demethylating agent, OR2100, and venetoclax for treatment of acute myeloid leukemia. Cancer Res Commun. (2023) 3:297–308. doi: 10.1158/2767-9764.CRC-22-0259 36860654 PMC9973401

[151] Molina JR Sun Y Protopopova M Gera S Bandi M Bristow C. An inhibitor of oxidative phosphorylation exploits cancer vulnerability. Nat Med. (2018) 24:1036–46. doi: 10.1038/s41591-018-0052-4 29892070

[152] Koptyra M Falinski R Nowicki MO Stoklosa T Majsterek I Nieborowska-Skorska M. BCR/ABL kinase induces self-mutagenesis via reactive oxygen species to encode imatinib resistance. Blood. (2006) 108:319–27. doi: 10.1182/blood-2005-07-2815 PMC1895841 16527898

[153] Sallmyr A Fan J Datta K Kim K-T Grosu D Shapiro P. Internal tandem duplication of FLT3 (FLT3/ITD) induces increased ROS production, DNA damage, and misrepair: implications for poor prognosis in AML. Blood. (2008) 111:3173–82. doi: 10.1182/blood-2007-05-092510 18192505

[154] Hole PS Pearn L Tonks AJ James PE Burnett AK Darley RL. Ras-induced reactive oxygen species promote growth factor–independent proliferation in human CD34+ hematopoietic progenitor cells. Blood. (2010) 115:1238–46. doi: 10.1182/blood-2009-06-222869 20007804

[155] Virtaneva K Wright FA Tanner SM Yuan B Lemon WJ Caligiuri MA. Expression profiling reveals fundamental biological differences in acute myeloid leukemia with isolated trisomy 8 and normal cytogenetics. Proc Natl Acad Sci U S A. (2001) 98:1124–9. doi: 10.1073/pnas.98.3.1124 PMC14719 11158605

[156] Schoch C Kohlmann A Schnittger S Brors B Dugas M Mergenthaler S. Acute myeloid leukemias with reciprocal rearrangements can be distinguished by specific gene expression profiles. Proc Natl Acad Sci U S A. (2002) 99:10008–13. doi: 10.1073/pnas.142103599 PMC126615 12105272

[157] Peterson LF Boyapati A Ahn E-Y Biggs JR Okumura AJ Lo M-C. Acute myeloid leukemia with the 8q22;21q22 translocation: secondary mutational events and alternative t(8;21) transcripts. Blood. (2007) 110:799–805. doi: 10.1182/blood-2006-11-019265 17412887 PMC1924771

[158] Höllein A Nadarajah N Meggendorfer M Jeromin S Kern W Haferlach C. Molecular characterization of AML with RUNX1-RUNX1T1 at diagnosis and relapse reveals net loss of co-mutations. HemaSphere. (2019) 3:e178. doi: 10.1097/HS9.0000000000000178 31723813 PMC6745937

[159] Weinstein JN Collisson EA Mills GB Shaw KRM Ozenberger BA Ellrott K. The Cancer Genome Atlas Pan-Cancer analysis project. Nat Genet. (2013) 45:1113–20. doi: 10.1038/ng.2764 PMC3919969 24071849

[160] Luo M Miao Y-R Ke Y-J Guo A-Y Zhang Q. A comprehensive landscape of transcription profiles and data resources for human leukemia. Blood Adv. (2023) 7:3435–49. doi: 10.1182/bloodadvances.2022008410 PMC10362280 36595475

[161] Petti AA Khan SM Xu Z Helton N Fronick CC Fulton R. Genetic and transcriptional contributions to relapse in normal karyotype acute myeloid leukemia. Blood Cancer Discovery. (2022) 3:32–49. doi: 10.1158/2643-3230.BCD-21-0050 35019859 PMC9924296

[162] Zavorka Thomas ME Jeon JY Talebi Z Buelow DR Silvaroli J Campbell MJ. Gilteritinib-induced upregulation of S100A9 is mediated through BCL6 in acute myeloid leukemia. Blood Adv. (2021) 5:5041–6. doi: 10.1182/bloodadvances.2021005614 PMC9153019 34614509

[163] Zhang L Zhou X Aryal S Veasey V Zhang P Li FJ. CRISPR screen of venetoclax response-associated genes identifies transcription factor ZNF740 as a key functional regulator. Cell Death Dis. (2024) 15:1–12. doi: 10.1038/s41419-024-06995-x 39191721 PMC11350041

[164] Hu T Zhang Y Yang T He Q Zhao M. LYPD3, a new biomarker and therapeutic target for acute myelogenous leukemia. Front Genet. (2022) 13:795820. doi: 10.3389/fgene.2022.795820 35360840 PMC8963240

[165] Nakano Y Naoe T Kiyoi H Kunishima S Minami S Miyawaki S. Poor clinical significance of p53 gene polymorphism in acute myeloid leukemia. Leuk Res. (2000) 24:349–52. doi: 10.1016/s0145-2126(99)00187-3 10713332

[166] Papaemmanuil E Gerstung M Bullinger L Gaidzik VI Paschka P Roberts ND. Genomic classification and prognosis in acute myeloid leukemia. N Engl J Med. (2016) 374:2209. doi: 10.1056/NEJMoa1516192 27276561 PMC4979995

[167] Sharon D Cathelin S Mirali S Di Trani JM Yanofsky DJ Keon KA. Inhibition of mitochondrial translation overcomes venetoclax resistance in AML through activation of the integrated stress response. Sci Transl Med. (2019) 11:eaax2863. doi: 10.1126/scitranslmed.aax2863 31666400

[168] Aasebø E Berven FS Bartaula-Brevik S Stokowy T Hovland R Vaudel M. Proteome and phosphoproteome changes associated with prognosis in acute myeloid leukemia. Cancers. (2020) 12:709. doi: 10.3390/cancers12030709 32192169 PMC7140113

[169] Hernandez-Valladares M Wangen R Aasebø E Reikvam H Berven FS Selheim F. Proteomic studies of primary acute myeloid leukemia cells derived from patients before and during disease-stabilizing treatment based on all-trans retinoic acid and valproic acid. Cancers. (2021)13:2143. doi: 10.3390/cancers13092143 33946813 PMC8125016

[170] Kramer MH Zhang Q Sprung R Day RB Erdmann-Gilmore P Li Y. Proteomic and phosphoproteomic landscapes of acute myeloid leukemia. Blood. (2022) 140:1533–48. doi: 10.1182/blood.2022016033 PMC9523374 35895896

[171] Seo W Yoo S Zhong Y Lee S-H Woo S-Y Choi H-S. Targeting ERRα promotes cytotoxic effects against acute myeloid leukemia through suppressing mitochondrial oxidative phosphorylation. J Hematol OncolJ Hematol Oncol. (2022) 15:156. doi: 10.1186/s13045-022-01372-7 36289517 PMC9597966

[172] Wu Y-T Tan H-L Shui G Bauvy C Huang Q Wenk MR. Dual role of 3-methyladenine in modulation of autophagy via different temporal patterns of inhibition on class I and III phosphoinositide 3-kinase. J Biol Chem. (2010) 285:10850–61. doi: 10.1074/jbc.M109.080796 PMC2856291 20123989

[173] KosChade SE Klann K Shaid S Vick B Stratmann JA Thölken M. Translatome proteomics identifies autophagy as a resistance mechanism to on-target FLT3 inhibitors in acute myeloid leukemia. Leukemia. (2022) 36:2396–407. doi: 10.1038/s41375-022-01678-y PMC9522593 35999260

[174] Jayavelu AK Wolf S Buettner F Alexe G Häupl B Comoglio F. The proteogenomic subtypes of acute myeloid leukemia. Cancer Cell. (2022) 40:301–317.e12. doi: 10.1016/j.ccell.2022.02.006 35245447 PMC12882723

[175] Sainero-Alcolado L Liaño-Pons J Ruiz-Pérez MV Arsenian-Henriksson M. Targeting mitochondrial metabolism for precision medicine in cancer. Cell Death Differ. (2022) 29:1304–17. doi: 10.1038/s41418-022-01022-y PMC9287557 35831624

[176] O’Brien C Ling T Berman JM Culp-Hill R Reisz JA Rondeau V. Simultaneous inhibition of Sirtuin 3 and cholesterol homeostasis targets acute myeloid leukemia stem cells by perturbing fatty acid β-oxidation and inducing lipotoxicity. Haematologica. (2023) 108:2343–57. doi: 10.3324/haematol.2022.281894 PMC10483359 37021547

